# Bayesian meta-analysis models for microarray data: a comparative study

**DOI:** 10.1186/1471-2105-8-80

**Published:** 2007-03-07

**Authors:** Erin M Conlon, Joon J Song, Anna Liu

**Affiliations:** 1 Department of Mathematics and Statistics, University of Massachusetts, Amherst, Massachusetts, USA; 2 Department of Mathematics, University of Arkansas, Fayetteville, Arkansas, USA

## Abstract

**Background:**

With the growing abundance of microarray data, statistical methods are increasingly needed to integrate results across studies. Two common approaches for meta-analysis of microarrays include either combining gene expression measures across studies or combining summaries such as p-values, probabilities or ranks. Here, we compare two Bayesian meta-analysis models that are analogous to these methods.

**Results:**

Two Bayesian meta-analysis models for microarray data have recently been introduced. The first model combines standardized gene expression measures across studies into an overall mean, accounting for inter-study variability, while the second combines probabilities of differential expression without combining expression values. Both models produce the gene-specific posterior probability of differential expression, which is the basis for inference. Since the standardized expression integration model includes inter-study variability, it may improve accuracy of results versus the probability integration model. However, due to the small number of studies typical in microarray meta-analyses, the variability between studies is challenging to estimate. The probability integration model eliminates the need to model variability between studies, and thus its implementation is more straightforward. We found in simulations of two and five studies that combining probabilities outperformed combining standardized gene expression measures for three comparison values: the percent of true discovered genes in meta-analysis versus individual studies; the percent of true genes omitted in meta-analysis versus separate studies, and the number of true discovered genes for fixed levels of Bayesian false discovery. We identified similar results when pooling two independent studies *of Bacillus subtilis*. We assumed that each study was produced from the same microarray platform with only two conditions: a treatment and control, and that the data sets were pre-scaled.

**Conclusion:**

The Bayesian meta-analysis model that combines probabilities across studies does not aggregate gene expression measures, thus an inter-study variability parameter is not included in the model. This results in a simpler modeling approach than aggregating expression measures, which accounts for variability across studies. The probability integration model identified more true discovered genes and fewer true omitted genes than combining expression measures, for our data sets.

## Background

Due to the growing accumulation of publicly available microarray data, it is increasingly important to develop methods to integrate findings across studies. Combining results will increase sample sizes and thus the power to detect differentially expressed genes. While meta-analysis has been used extensively in medical and public health applications, it has only recently been developed for microarray studies [1-15]. The two primary methods for data integration consist of either combining gene expression measures across studies or combining summary measures of expression such as *p*-values, probabilities or ranks. In the first approach, Wang *et al*. [1] used a weighted average procedure to combine standardized mean expression differences across three independent studies. Through this method they identified genes that were consistently differentially expressed between leukemia and normal B cells. Choi *et al*. [2] and Stevens and Doerge [3] combined standardized gene effects into an overall mean effect using statistical modeling. The models accounted for different sources of variation in microarray studies, including differences between studies. Since inter-study variability was assumed to be constant for each gene, the uncertainty of this parameter was not included in the subsequent analyses (this refers only to the *non-Bayesian *model of Choi *et al*. [2]). Hu *et al*. [4] extended the method of Choi *et al*. [2] by incorporating an individual study quality index for each gene into the effect size estimate. The authors combined two lung cancer data sets and demonstrated that their method identified more differentially expressed genes than previous analyses. Other investigators combined "raw" gene expression data rather than gene effects when the data was comparable across studies. Examples include Morris *et al*. [5], which combined Affymetrix studies by creating new probesets across arrays, and Park *et al*. [6], which integrated log-expression ratios in an ANOVA meta-analysis model for cDNA microarrays.

Due to the difficulty in comparing cross-laboratory and cross-platform expression measures, several microarray meta-analysis methods have combined summary measures of expression rather than expression measures themselves. Rhodes *et al*. [7] calculated *p*-values in individual lung cancer studies and aggregated the *p*-values to provide an overall estimate of gene significance. Parmigiani *et al*. [9] introduced an integrative correlation approach that identified genes with consistent expression patterns across multiple platforms. Other approaches convert gene expression values within each study to rank orderings or probabilities of expression. The transformed data is then aggregated across studies to identify disease marker genes or prognostic signatures (Shen *et al*. [10], Xu *et al*. [11], Warnat *et al*. [12]).

Recently, Bayesian meta-analysis models have been introduced that are analogous to the classical methods described above. Bayesian approaches have several advantages over traditional methods and have been used widely in individual microarray studies [16-18]; in particular the discrete mixture model has been developed extensively [19-30]. Bayesian models are well-suited to the small sample sizes of microarray studies since they borrow information from all genes to estimate model parameters. They also provide a framework for incorporating all available information in a systematic manner, and explicitly include model and parameter variability. A third important benefit of the Bayesian approach is that a predictive distribution for future data is produced (Stangl and Berry [31], Tweedie *et al*. [32]). Bayesian models can also handle the large amounts of missing data inherent in microarray studies relatively easily.

The two primary Bayesian approaches to meta-analysis for microarray studies correspond to the traditional approaches: one combines standardized gene effects and the other combines probabilities, as follows. Choi *et al*. [2] introduced the first Bayesian meta-analysis model for microarray data, which integrated standardized gene effects in individual studies into an overall mean effect. Inter-study variability was included in the model with an associated uninformative prior distribution. This type of model, termed *hierarchical Bayesian random effects*, has been used broadly in non-microarray contexts (see, for example, DuMouchel and Harris [33], Smith *et al*. [34], Tweedie *et al*. [32], Normand [35], DuMouchel and Normand [36], Pauler and Wakefield [37], Sargent *et al*. [38], Gelman *et al*. [39]). The hierarchical Bayesian random effects meta-analysis model has several favorable features: it provides an overall effect size among all studies, and it accounts for inter-study variability, which may improve accuracy of results. However, many microarray meta-analyses include a small number of studies, e.g. between two and four studies [1,2,4-7,9-11,13-15]. Due to these typical small study numbers, the inter-study variability is challenging to model. An uninformative prior distribution for this parameter may not provide enough information, and thus more informative priors need to be considered. Alternatively, Conlon *et al*. [15] introduced a Bayesian meta-analysis model that combined study probabilities rather than gene effect levels, eliminating the need to estimate inter-study variability. The model produced the overall gene-specific posterior probability of differential expression while incorporating several sources of data replication. Here, we compare two Bayesian meta-analysis models: the standardized expression integration model and the probability integration model of Conlon *et al*. [15]. The standardized expression integration model is similar in approach to hierarchical Bayesian random effects models (such as that of Choi *et al*. [2]) in that mean values are combined across studies to provide an overall mean value, with inter-study variability included as a parameter in the model with an associated prior distribution. However, instead of combining effect sizes as in Choi *et al*., we combine standardized gene expression values, since our data is assumed to be from the same platform and comparable across studies (for further detail, see Background: Background on effect sizes and standardized gene expression levels). In simulations, we illustrate that combining probabilities improves performance versus combining standardized gene expression levels based on three comparison measures: the increase in true discovered genes in meta-analysis versus separate studies, the decrease in true omitted genes in meta-analysis versus individual studies, and the number of true genes identified for fixed levels of Bayesian false discovery. Our findings are similar when analyzing biological data in two studies *of Bacillus *(*B*.) *subtilis*.

While many meta-analytic methods incorporate data across multiple microarray platforms, several recent reports have shown the difficulty in such approaches. Kuo *et al*. [40] and Jarvinen *et al*. [41] both worked with cell lines and concluded that combining data across cDNA and oligonucleotide platforms was not reliable. Mah *et al*. [42] found that there is only moderate overlap in gene expression levels between cDNA and oligonucleotide platforms. Due to these findings, we focus on meta-analysis for one platform, cDNA microarrays.

### Background on effect sizes and standardized gene expression levels

Traditional random effects models and hierarchical Bayesian random effects models first summarize the data from two conditions into study-specific mean effects and associated variances; the models use these summary effect measures rather than the individual data values (i.e. "raw" data). For instance, the hierarchical Bayesian random effects model of Choi *et al*. [2] used the well-studied estimator of Hedges and Olkin [43], which estimates the effect size as the difference of the means of two groups divided by the pooled standard deviation. The estimator is sample-size adjusted to obtain an unbiased effect size; the associated variance is also based on Hedges and Olkin's work. However, Hedges and Olkin's method requires two separate groups of data with only one level of replication, which is not always available. For one sample data with replicate slides within repeated experiments, the Hedges and Olkin effect size estimates do not apply. For these reasons, for our one-sample cDNA microarray studies with multiple sources of replication, we use the standardized logarithms of the red/green (Cy5/Cy3) fluorescent intensity ratios in our meta-analysis models. We use log-ratios since they create more symmetric distributions and stabilize the variances (Dudoit *et al*. [44]). The log-expression ratios are standardized so that each slide has zero mean and unit standard deviation (see also Shen *et al*. [10] and Dominici and Parmigiani [45]). For further background on cDNA microarrays, see [46-49].

## Results and discussion

### Bayesian standardized expression integration meta-analysis model

Researchers often conduct multiple independent microarray studies for the same biological system. For example, Eichenberger *et al*. [50] designed two studies to identify genes under the control of a primary transcription factor sigma E (*σ*^*E*^) in *B. subtilis*. The first study was a mutation of *σ*^*E *^and the second was an overexpression of *σ*^*E *^(referred to as the mutant and induction studies, respectively; for details of the data sets, see Methods: Biological data). Thus, genes that were overexpressed in one study should be underexpressed in the other. Combining results of the two studies in a meta-analysis will increase sample size and help more precisely identify target genes. In the Bayesian standardized expression integration model, expression levels are smoothed across studies to produce an overall expression measure for each gene. This model assumes that the standardized expression means are not the same in each study, but that each study-specific mean is a random sample from a common population distribution. Inter-study variability is included as a parameter in the model. More specifically,

yjgse|ξjge~N(ξjge,φjg2), j=1,...,J; g=1,...,G; s=1,...,Se; e=1,...,Eξjge|θjg~N(θjg,σjg2), j=1,...,J; g=1,...,G; e=1,...,Eθjg|μg~N(μg,τg2), j=1,...,J; g=1,...,Gμg|Ig=0~N(0,ηg02)μg|Ig=1~N(0,c×ηg02)Ig~Bernoulli(p)p~Uniform(0,1),     (1)
 MathType@MTEF@5@5@+=feaafiart1ev1aaatCvAUfKttLearuWrP9MDH5MBPbIqV92AaeXatLxBI9gBaebbnrfifHhDYfgasaacH8akY=wiFfYdH8Gipec8Eeeu0xXdbba9frFj0=OqFfea0dXdd9vqai=hGuQ8kuc9pgc9s8qqaq=dirpe0xb9q8qiLsFr0=vr0=vr0dc8meaabaqaciaacaGaaeqabaqabeGadaaakeaafaqaaeWbdaaaaeaacqWG5bqEdaWgaaWcbaGaemOAaOMaem4zaCMaem4CamNaemyzaugabeaakiabcYha8HGaciab=57a4naaBaaaleaacqWGQbGAcqWGNbWzcqWGLbqzaeqaaaGcbaGaeiOFa4habaGaeeOta4KaeiikaGIae8NVdG3aaSbaaSqaaiabdQgaQjabdEgaNjabdwgaLbqabaGccqGGSaalcqWFgpGzdaqhaaWcbaGaemOAaOMaem4zaCgabaGaeGOmaidaaOGaeiykaKIaeiilaWIaeeiiaaIaemOAaOMaeyypa0JaeGymaeJaeiilaWIaeiOla4IaeiOla4IaeiOla4IaeiilaWIaemOsaOKaei4oaSJaeeiiaaIaem4zaCMaeyypa0JaeGymaeJaeiilaWIaeiOla4IaeiOla4IaeiOla4IaeiilaWIaem4raCKaei4oaSJaeeiiaaIaem4CamNaeyypa0JaeGymaeJaeiilaWIaeiOla4IaeiOla4IaeiOla4IaeiilaWIaem4uam1aaSbaaSqaaiabdwgaLbqabaGccqGG7aWocqqGGaaicqWGLbqzcqGH9aqpcqaIXaqmcqGGSaalcqGGUaGlcqGGUaGlcqGGUaGlcqGGSaalcqWGfbqraeaacqWF+oaEdaWgaaWcbaGaemOAaOMaem4zaCMaemyzaugabeaakiabcYha8jab=H7aXnaaBaaaleaacqWGQbGAcqWGNbWzaeqaaaGcbaGaeiOFa4habaGaeeOta4KaeiikaGIae8hUde3aaSbaaSqaaiabdQgaQjabdEgaNbqabaGccqGGSaalcqWFdpWCdaqhaaWcbaGaemOAaOMaem4zaCgabaGaeGOmaidaaOGaeiykaKIaeiilaWIaeeiiaaIaemOAaOMaeyypa0JaeGymaeJaeiilaWIaeiOla4IaeiOla4IaeiOla4IaeiilaWIaemOsaOKaei4oaSJaeeiiaaIaem4zaCMaeyypa0JaeGymaeJaeiilaWIaeiOla4IaeiOla4IaeiOla4IaeiilaWIaem4raCKaei4oaSJaeeiiaaIaemyzauMaeyypa0JaeGymaeJaeiilaWIaeiOla4IaeiOla4IaeiOla4IaeiilaWIaemyraueabaGae8hUde3aaSbaaSqaaiabdQgaQjabdEgaNbqabaGccqGG8baFcqWF8oqBdaWgaaWcbaGaem4zaCgabeaaaOqaaiabc6ha+bqaaiabb6eaojabcIcaOiab=X7aTnaaBaaaleaacqWGNbWzaeqaaOGaeiilaWIae8hXdq3aa0baaSqaaiabdEgaNbqaaiabikdaYaaakiabcMcaPiabcYcaSiabbccaGiabdQgaQjabg2da9iabigdaXiabcYcaSiabc6caUiabc6caUiabc6caUiabcYcaSiabdQeakjabcUda7iabbccaGiabdEgaNjabg2da9iabigdaXiabcYcaSiabc6caUiabc6caUiabc6caUiabcYcaSiabdEeahbqaaiab=X7aTnaaBaaaleaacqWGNbWzaeqaaOGaeiiFaWNaemysaK0aaSbaaSqaaiabdEgaNbqabaGccqGH9aqpcqaIWaamaeaacqGG+bGFaeaacqqGobGtcqGGOaakcqaIWaamcqGGSaalcqWF3oaAdaqhaaWcbaGaem4zaCMaeGimaadabaGaeGOmaidaaOGaeiykaKcabaGae8hVd02aaSbaaSqaaiabdEgaNbqabaGccqGG8baFcqWGjbqsdaWgaaWcbaGaem4zaCgabeaakiabg2da9iabigdaXaqaaiabc6ha+bqaaiabb6eaojabcIcaOiabicdaWiabcYcaSiabdogaJjabgEna0kab=D7aOnaaDaaaleaacqWGNbWzcqaIWaamaeaacqaIYaGmaaGccqGGPaqkaeaacqWGjbqsdaWgaaWcbaGaem4zaCgabeaaaOqaaiabc6ha+bqaaiabbkeacjabbwgaLjabbkhaYjabb6gaUjabb+gaVjabbwha1jabbYgaSjabbYgaSjabbMgaPjabcIcaOiabdchaWjabcMcaPaqaaiabdchaWbqaaiabc6ha+bqaaiabbwfavjabb6gaUjabbMgaPjabbAgaMjabb+gaVjabbkhaYjabb2gaTjabcIcaOiabicdaWiabcYcaSiabigdaXiabcMcaPiabcYcaSaaacaWLjaGaaCzcamaabmaabaGaeGymaedacaGLOaGaayzkaaaaaa@33E7@

for *J *independent studies. Here, *y*_*jgse *_are the observed microarray data, and are the normalized log-expression ratios for study *j*, gene *g*, slide *s*, and experiment *e*. For cDNA microarrays, the expression ratio is the ratio of fluorescent intensity levels for the red and green-labeled mRNA (Cy5 and Cy3) samples, or treatment and control. We standardized the *y*_*jgse *_values so that each slide has zero mean and unit standard deviation (see also [10,45]). In this model, we take into account that the *y*_*jgse *_are affected by variability due to slides, experiments (cultures) and studies. In individual studies, *y*_*jgse *_is a sample from a normal distribution of the slide values within the same experiment for gene *g*. We represent this in the model as *y*_*jgse *_~ N(*ξ*_*jge*_, φjg2
 MathType@MTEF@5@5@+=feaafiart1ev1aaatCvAUfKttLearuWrP9MDH5MBPbIqV92AaeXatLxBI9gBaebbnrfifHhDYfgasaacH8akY=wiFfYdH8Gipec8Eeeu0xXdbba9frFj0=OqFfea0dXdd9vqai=hGuQ8kuc9pgc9s8qqaq=dirpe0xb9q8qiLsFr0=vr0=vr0dc8meaabaqaciaacaGaaeqabaqabeGadaaakeaaiiGacqWFgpGzdaqhaaWcbaGaemOAaOMaem4zaCgabaGaeGOmaidaaaaa@323F@) where *ξ*_*jge *_is the mean among all slide values of an experiment for gene *g*. The parameter φjg2
 MathType@MTEF@5@5@+=feaafiart1ev1aaatCvAUfKttLearuWrP9MDH5MBPbIqV92AaeXatLxBI9gBaebbnrfifHhDYfgasaacH8akY=wiFfYdH8Gipec8Eeeu0xXdbba9frFj0=OqFfea0dXdd9vqai=hGuQ8kuc9pgc9s8qqaq=dirpe0xb9q8qiLsFr0=vr0=vr0dc8meaabaqaciaacaGaaeqabaqabeGadaaakeaaiiGacqWFgpGzdaqhaaWcbaGaemOAaOMaem4zaCgabaGaeGOmaidaaaaa@323F@ represents the variability of the slide value distribution for each gene. The within-experiment mean *ξ*_*jge *_is a sampling from a normal distribution of experiment values; we denote this as *ξ*_*jge *_~ N(*θ*_*jg*_, σjg2
 MathType@MTEF@5@5@+=feaafiart1ev1aaatCvAUfKttLearuWrP9MDH5MBPbIqV92AaeXatLxBI9gBaebbnrfifHhDYfgasaacH8akY=wiFfYdH8Gipec8Eeeu0xXdbba9frFj0=OqFfea0dXdd9vqai=hGuQ8kuc9pgc9s8qqaq=dirpe0xb9q8qiLsFr0=vr0=vr0dc8meaabaqaciaacaGaaeqabaqabeGadaaakeaaiiGacqWFdpWCdaqhaaWcbaGaemOAaOMaem4zaCgabaGaeGOmaidaaaaa@3249@). Here, *θ*_*jg *_is the study-specific mean log-expression ratio of gene *g*, and σjg2
 MathType@MTEF@5@5@+=feaafiart1ev1aaatCvAUfKttLearuWrP9MDH5MBPbIqV92AaeXatLxBI9gBaebbnrfifHhDYfgasaacH8akY=wiFfYdH8Gipec8Eeeu0xXdbba9frFj0=OqFfea0dXdd9vqai=hGuQ8kuc9pgc9s8qqaq=dirpe0xb9q8qiLsFr0=vr0=vr0dc8meaabaqaciaacaGaaeqabaqabeGadaaakeaaiiGacqWFdpWCdaqhaaWcbaGaemOAaOMaem4zaCgabaGaeGOmaidaaaaa@3249@ represents the variability across experiments. In turn, the study-specific mean *θ*_*jg *_is a sampling from a normal distribution of study values. Thus, *θ*_*jg *_~ N(*μ*_*g*_, τg2
 MathType@MTEF@5@5@+=feaafiart1ev1aaatCvAUfKttLearuWrP9MDH5MBPbIqV92AaeXatLxBI9gBaebbnrfifHhDYfgasaacH8akY=wiFfYdH8Gipec8Eeeu0xXdbba9frFj0=OqFfea0dXdd9vqai=hGuQ8kuc9pgc9s8qqaq=dirpe0xb9q8qiLsFr0=vr0=vr0dc8meaabaqaciaacaGaaeqabaqabeGadaaakeaaiiGacqWFepaDdaqhaaWcbaGaem4zaCgabaGaeGOmaidaaaaa@30EE@), where *μ*_*g *_represents the overall mean log-expression ratio across studies, and τg2
 MathType@MTEF@5@5@+=feaafiart1ev1aaatCvAUfKttLearuWrP9MDH5MBPbIqV92AaeXatLxBI9gBaebbnrfifHhDYfgasaacH8akY=wiFfYdH8Gipec8Eeeu0xXdbba9frFj0=OqFfea0dXdd9vqai=hGuQ8kuc9pgc9s8qqaq=dirpe0xb9q8qiLsFr0=vr0=vr0dc8meaabaqaciaacaGaaeqabaqabeGadaaakeaaiiGacqWFepaDdaqhaaWcbaGaem4zaCgabaGaeGOmaidaaaaa@30EE@ is the variability across studies. The *μ*_*g *_values are assumed to be normally distributed with a mean of zero with a small variance for non-differentially expressed genes, and with a large variance for differentially expressed genes. Note that only *y*_*jgse *_values are observed data, while the remaining parameters are unobserved. Based on the overall mean value *μ*_*g*_, the model produces the posterior distribution for *I*_*g*_, which is used to calculate the probability of differential expression *D*_*g *_= Prob(*I*_*g *_= 1 | data). More specifically, *I*_*g *_~ Bernoulli(*p*) is the indicator variable for differential expression of gene *g *corresponding to *μ*_*g *_≠ 0, and *p *is the fraction of expressed genes. Thus, Prob(*I*_*g *_= 1) = *p*, where

Ig={0 if μg=01 if μg≠0
 MathType@MTEF@5@5@+=feaafiart1ev1aaatCvAUfKttLearuWrP9MDH5MBPbIqV92AaeXatLxBI9gBaebbnrfifHhDYfgasaacH8akY=wiFfYdH8Gipec8Eeeu0xXdbba9frFj0=OqFfea0dXdd9vqai=hGuQ8kuc9pgc9s8qqaq=dirpe0xb9q8qiLsFr0=vr0=vr0dc8meaabaqaciaacaGaaeqabaqabeGadaaakeaacqWGjbqsdaWgaaWcbaGaem4zaCgabeaakiabg2da9maaceqabaqbaeqabiqaaaqaaiabicdaWiabbccaGiabbMgaPjabbAgaMjabbccaGGGaciab=X7aTnaaBaaaleaacqWGNbWzaeqaaOGaeyypa0JaeGimaadabaGaeGymaeJaeeiiaaIaeeyAaKMaeeOzayMaeeiiaaIae8hVd02aaSbaaSqaaiabdEgaNbqabaGccqGHGjsUcqaIWaamaaaacaGL7baaaaa@4705@

With this model, genes are divided into two groups, differentially expressed (*I*_*g *_= 1) and non-expressed (*I*_*g *_= 0), with probabilities *p *and (1-*p*), respectively. For each gene, the posterior probability of differential expression over all studies, *D*_*g *_= Prob(*I*_*g *_= 1 | data), is produced, and we compare results based on *D*_*g*_. In assigning prior distributions, when *I*_*g *_= 0, the *μ*_*g *_are assumed to be normally distributed with mean zero and small variance ηg02
 MathType@MTEF@5@5@+=feaafiart1ev1aaatCvAUfKttLearuWrP9MDH5MBPbIqV92AaeXatLxBI9gBaebbnrfifHhDYfgasaacH8akY=wiFfYdH8Gipec8Eeeu0xXdbba9frFj0=OqFfea0dXdd9vqai=hGuQ8kuc9pgc9s8qqaq=dirpe0xb9q8qiLsFr0=vr0=vr0dc8meaabaqaciaacaGaaeqabaqabeGadaaakeaaiiGacqWF3oaAdaqhaaWcbaGaem4zaCMaeGimaadabaGaeGOmaidaaaaa@31C3@ ; when *I*_*g *_= 1, the *μ*_*g *_are assumed to be normally distributed with mean zero and large variance (*c *× ηg02
 MathType@MTEF@5@5@+=feaafiart1ev1aaatCvAUfKttLearuWrP9MDH5MBPbIqV92AaeXatLxBI9gBaebbnrfifHhDYfgasaacH8akY=wiFfYdH8Gipec8Eeeu0xXdbba9frFj0=OqFfea0dXdd9vqai=hGuQ8kuc9pgc9s8qqaq=dirpe0xb9q8qiLsFr0=vr0=vr0dc8meaabaqaciaacaGaaeqabaqabeGadaaakeaaiiGacqWF3oaAdaqhaaWcbaGaem4zaCMaeGimaadabaGaeGOmaidaaaaa@31C3@).

The inter-study variability parameter τg2
 MathType@MTEF@5@5@+=feaafiart1ev1aaatCvAUfKttLearuWrP9MDH5MBPbIqV92AaeXatLxBI9gBaebbnrfifHhDYfgasaacH8akY=wiFfYdH8Gipec8Eeeu0xXdbba9frFj0=OqFfea0dXdd9vqai=hGuQ8kuc9pgc9s8qqaq=dirpe0xb9q8qiLsFr0=vr0=vr0dc8meaabaqaciaacaGaaeqabaqabeGadaaakeaaiiGacqWFepaDdaqhaaWcbaGaem4zaCgabaGaeGOmaidaaaaa@30EE@ influences the results to a large degree and requires careful consideration. We detail several specifications for the prior distribution of τg2
 MathType@MTEF@5@5@+=feaafiart1ev1aaatCvAUfKttLearuWrP9MDH5MBPbIqV92AaeXatLxBI9gBaebbnrfifHhDYfgasaacH8akY=wiFfYdH8Gipec8Eeeu0xXdbba9frFj0=OqFfea0dXdd9vqai=hGuQ8kuc9pgc9s8qqaq=dirpe0xb9q8qiLsFr0=vr0=vr0dc8meaabaqaciaacaGaaeqabaqabeGadaaakeaaiiGacqWFepaDdaqhaaWcbaGaem4zaCgabaGaeGOmaidaaaaa@30EE@ in the sections: Two-study simulation results; and Methods: Standardized expression integration model: prior distributions for inter-study variation.

We assign conjugate scaled inverse chi-squared prior distributions to the slide and experiment variation parameters, φjg2
 MathType@MTEF@5@5@+=feaafiart1ev1aaatCvAUfKttLearuWrP9MDH5MBPbIqV92AaeXatLxBI9gBaebbnrfifHhDYfgasaacH8akY=wiFfYdH8Gipec8Eeeu0xXdbba9frFj0=OqFfea0dXdd9vqai=hGuQ8kuc9pgc9s8qqaq=dirpe0xb9q8qiLsFr0=vr0=vr0dc8meaabaqaciaacaGaaeqabaqabeGadaaakeaaiiGacqWFgpGzdaqhaaWcbaGaemOAaOMaem4zaCgabaGaeGOmaidaaaaa@323F@ and σjg2
 MathType@MTEF@5@5@+=feaafiart1ev1aaatCvAUfKttLearuWrP9MDH5MBPbIqV92AaeXatLxBI9gBaebbnrfifHhDYfgasaacH8akY=wiFfYdH8Gipec8Eeeu0xXdbba9frFj0=OqFfea0dXdd9vqai=hGuQ8kuc9pgc9s8qqaq=dirpe0xb9q8qiLsFr0=vr0=vr0dc8meaabaqaciaacaGaaeqabaqabeGadaaakeaaiiGacqWFdpWCdaqhaaWcbaGaemOAaOMaem4zaCgabaGaeGOmaidaaaaa@3249@. The scale parameters are derived from the data, by pooling information from all genes (similar to Tseng *et al*. [17], Gottardo *et al*. [23], Lönnstedt and Speed [24]; see Methods). The prior framework for a single study is similar to Gottardo *et al*. [23] except that we calculate a posterior distribution for *p *rather than using an iterative algorithm to estimate *p*. Our data also has one more level of replication than that of Gottardo *et al*. [23], i.e. repeated slides within repeated experiments. Our hierarchical Gaussian structure for one study also resembles the Bayesian ANOVA models (BAM) of Ishwaran and Rao [29,30]. BAM restructures the problem of identifying overexpressed genes as a variable selection procedure and uses a Bayesian model designed toward selective shrinkage. The difference between our model in a single study context and BAM is that we have one-sample data, while BAM models are tailored to a two-sample format; our data also has more levels of replication.

Each study is assumed to contain only two conditions: a treatment and a control. We simulate posterior distributions for each parameter using a Markov chain Monte Carlo (MCMC) implementation of the model [51]. See the Methods section for more details of the prior distributions and the MCMC procedure.

### Bayesian probability integration meta-analysis model

The Bayesian model to combine probabilities was introduced in Conlon *et al*. [15] and is similar to the Bayesian standardized expression integration model, except that the mean expression levels of each study are not combined and thus inter-study variability is not modeled. We call this the probability integration model because, for each gene, it calculates the overall probability of differential expression given the data of each separate study; this effectively smoothes the probability of differential expression across studies. This differs from the standardized expression integration model, which first calculates an overall mean gene expression measure and determines the probability of differential expression given the estimated mean. We specify the probability integration model as follows:

yjgse|ξjge~N(ξjge,φjg2), j=1,...,J; g=1,...,G; e=1,...,E; s=1,...,Seξjge|θjg~N(θjg,σjg2), j=1,...,J; g=1,...,G; e=1,...,Eθjg|Ig=0~N(0,ηjg02)θjg|Ig=1~N(0,cj×ηjg02)Ig~Bernoulli(p)p~Uniform(0,1).     (2)
 MathType@MTEF@5@5@+=feaafiart1ev1aaatCvAUfKttLearuWrP9MDH5MBPbIqV92AaeXatLxBI9gBaebbnrfifHhDYfgasaacH8akY=wiFfYdH8Gipec8Eeeu0xXdbba9frFj0=OqFfea0dXdd9vqai=hGuQ8kuc9pgc9s8qqaq=dirpe0xb9q8qiLsFr0=vr0=vr0dc8meaabaqaciaacaGaaeqabaqabeGadaaakeaafaqaaeGbdaaaaeaacqWG5bqEdaWgaaWcbaGaemOAaOMaem4zaCMaem4CamNaemyzaugabeaakiabcYha8HGaciab=57a4naaBaaaleaacqWGQbGAcqWGNbWzcqWGLbqzaeqaaaGcbaGaeiOFa4habaGaeeOta4KaeiikaGIae8NVdG3aaSbaaSqaaiabdQgaQjabdEgaNjabdwgaLbqabaGccqGGSaalcqWFgpGzdaqhaaWcbaGaemOAaOMaem4zaCgabaGaeGOmaidaaOGaeiykaKIaeiilaWIaeeiiaaIaemOAaOMaeyypa0JaeGymaeJaeiilaWIaeiOla4IaeiOla4IaeiOla4IaeiilaWIaemOsaOKaei4oaSJaeeiiaaIaem4zaCMaeyypa0JaeGymaeJaeiilaWIaeiOla4IaeiOla4IaeiOla4IaeiilaWIaem4raCKaei4oaSJaeeiiaaIaemyzauMaeyypa0JaeGymaeJaeiilaWIaeiOla4IaeiOla4IaeiOla4IaeiilaWIaemyrauKaei4oaSJaeeiiaaIaem4CamNaeyypa0JaeGymaeJaeiilaWIaeiOla4IaeiOla4IaeiOla4IaeiilaWIaem4uam1aaSbaaSqaaiabdwgaLbqabaaakeaacqWF+oaEdaWgaaWcbaGaemOAaOMaem4zaCMaemyzaugabeaakiabcYha8jab=H7aXnaaBaaaleaacqWGQbGAcqWGNbWzaeqaaaGcbaGaeiOFa4habaGaeeOta4KaeiikaGIae8hUde3aaSbaaSqaaiabdQgaQjabdEgaNbqabaGccqGGSaalcqWFdpWCdaqhaaWcbaGaemOAaOMaem4zaCgabaGaeGOmaidaaOGaeiykaKIaeiilaWIaeeiiaaIaemOAaOMaeyypa0JaeGymaeJaeiilaWIaeiOla4IaeiOla4IaeiOla4IaeiilaWIaemOsaOKaei4oaSJaeeiiaaIaem4zaCMaeyypa0JaeGymaeJaeiilaWIaeiOla4IaeiOla4IaeiOla4IaeiilaWIaem4raCKaei4oaSJaeeiiaaIaemyzauMaeyypa0JaeGymaeJaeiilaWIaeiOla4IaeiOla4IaeiOla4IaeiilaWIaemyraueabaGae8hUde3aaSbaaSqaaiabdQgaQjabdEgaNbqabaGccqGG8baFcqWGjbqsdaWgaaWcbaGaem4zaCgabeaakiabg2da9iabicdaWaqaaiabc6ha+bqaaiabb6eaojabcIcaOiabicdaWiabcYcaSiab=D7aOnaaDaaaleaacqWGQbGAcqWGNbWzcqaIWaamaeaacqaIYaGmaaGccqGGPaqkaeaacqWF4oqCdaWgaaWcbaGaemOAaOMaem4zaCgabeaakiabcYha8jabdMeajnaaBaaaleaacqWGNbWzaeqaaOGaeyypa0JaeGymaedabaGaeiOFa4habaGaeeOta4KaeiikaGIaeGimaaJaeiilaWIaem4yam2aaSbaaSqaaiabdQgaQbqabaGccqGHxdaTcqWF3oaAdaqhaaWcbaGaemOAaOMaem4zaCMaeGimaadabaGaeGOmaidaaOGaeiykaKcabaGaemysaK0aaSbaaSqaaiabdEgaNbqabaaakeaacqGG+bGFaeaacqqGcbGqcqqGLbqzcqqGYbGCcqqGUbGBcqqGVbWBcqqG1bqDcqqGSbaBcqqGSbaBcqqGPbqAcqGGOaakcqWGWbaCcqGGPaqkaeaacqWGWbaCaeaacqGG+bGFaeaacqqGvbqvcqqGUbGBcqqGPbqAcqqGMbGzcqqGVbWBcqqGYbGCcqqGTbqBcqGGOaakcqaIWaamcqGGSaalcqaIXaqmcqGGPaqkcqGGUaGlaaGaaCzcaiaaxMaadaqadaqaaiabikdaYaGaayjkaiaawMcaaaaa@0FC8@

The parameters common to both Models (1) and (2) are as defined for Model (1) (see also Conlon *et al*. [15]). The *y*_*jgse *_are again the observed microarray data, and are the normalized log-expression ratios for study *j*, gene *g*, slide *s*, and experiment *e*. In this model, we again take into account that the *y*_*jgse *_are affected by variability due to slides and experiments, and the *y*_*jgse *_values are standardized to have zero mean and unit standard deviation. The *θ*_*jg *_is again the study-specific mean log-expression ratio of gene *g*. However, rather than modeling the study-specific mean *θ*_*jg *_as a sampling from a normal distribution of study values, this model treats each study separately, and does not combine the mean values from each study into one mean value. Thus, an overall mean value *μ*_*g *_is not produced for this model. The gene-specific posterior probability of differential expression is again produced; however, this probability is based upon the separate mean levels of each study rather than an overall mean level, as in Model (1). Note that the differences between Model (1) and Model (2) occur at the inter-study level; the models are the same for a single study. The MCMC implementation is similar to that for Model (1), with details in the Methods section. We compare Models (1) and (2) using integration-driven discovery rates, integration-driven revision rates and Bayesian false discovery rates, defined in the following.

### Integration-driven discovery and revision

Choi *et al*. [2] define the integration-driven discovery rate (IDR) as the proportion of genes that are identified as differentially expressed in the meta-analysis that were not identified in any of the individual studies alone. IDR represents the increase in information based on combining studies versus individual studies. For our models, for a given threshold of posterior probability of differential expression, *γ, *genes are identified as differentially expressed if (*D*_*g *_≥ *γ*). The IDR is defined as the percent of differentially expressed genes in the meta-analysis that are not differentially expressed in any of the individual analyses:

IDR(γ)=#genes [(Dg≥γ) in meta-analysis] and [(Dg<γ) in all individual studies]#genes [(Dg≥γ) in meta-analysis].
 MathType@MTEF@5@5@+=feaafiart1ev1aaatCvAUfKttLearuWrP9MDH5MBPbIqV92AaeXatLxBI9gBaebbnrfifHhDYfgasaacH8akY=wiFfYdH8Gipec8Eeeu0xXdbba9frFj0=OqFfea0dXdd9vqai=hGuQ8kuc9pgc9s8qqaq=dirpe0xb9q8qiLsFr0=vr0=vr0dc8meaabaqaciaacaGaaeqabaqabeGadaaakeaacqqGjbqscqqGebarcqqGsbGucqGGOaakiiGacqWFZoWzcqGGPaqkcqGH9aqpdaWcaaqaaiabcocaJiabbEgaNjabbwgaLjabb6gaUjabbwgaLjabbohaZjabbccaGiabcUfaBjabcIcaOiabdseaenaaBaaaleaacqWGNbWzaeqaaOGaeyyzImRae83SdCMaeiykaKIaeeiiaaIaeeyAaKMaeeOBa4MaeeiiaaIaeeyBa0MaeeyzauMaeeiDaqNaeeyyaeMaeeyla0IaeeyyaeMaeeOBa4MaeeyyaeMaeeiBaWMaeeyEaKNaee4CamNaeeyAaKMaee4CamNaeiyxa0LaeeiiaaIaeeyyaeMaeeOBa4MaeeizaqMaeeiiaaIaei4waSLaeiikaGIaemiraq0aaSbaaSqaaiabdEgaNbqabaGccqGH8aapcqWFZoWzcqGGPaqkcqqGGaaicqqGPbqAcqqGUbGBcqqGGaaicqqGHbqycqqGSbaBcqqGSbaBcqqGGaaicqqGPbqAcqqGUbGBcqqGKbazcqqGPbqAcqqG2bGDcqqGPbqAcqqGKbazcqqG1bqDcqqGHbqycqqGSbaBcqqGGaaicqqGZbWCcqqG0baDcqqG1bqDcqqGKbazcqqGPbqAcqqGLbqzcqqGZbWCcqGGDbqxaeaacqGGJaWicqqGNbWzcqqGLbqzcqqGUbGBcqqGLbqzcqqGZbWCcqqGGaaicqGGBbWwcqGGOaakcqWGebardaWgaaWcbaGaem4zaCgabeaakiabgwMiZkab=n7aNjabcMcaPiabbccaGiabbMgaPjabb6gaUjabbccaGiabb2gaTjabbwgaLjabbsha0jabbggaHjabb2caTiabbggaHjabb6gaUjabbggaHjabbYgaSjabbMha5jabbohaZjabbMgaPjabbohaZjabb2faDbaacqGGUaGlaaa@B5D3@

In simulations, we define true genes as genes that were simulated to be differentially expressed. The corresponding true integration-driven discovery rate, *t*IDR, is the percent of true genes discovered in the meta-analysis that were not discovered in any of the individual analyses:

tIDR(γ)=#true genes [(Dg≥γ) in meta-analysis] and [(Dg<γ) in all individual studies]#true genes [(Dg≥γ) in meta-analysis].
 MathType@MTEF@5@5@+=feaafiart1ev1aaatCvAUfKttLearuWrP9MDH5MBPbIqV92AaeXatLxBI9gBaebbnrfifHhDYfgasaacH8akY=wiFfYdH8Gipec8Eeeu0xXdbba9frFj0=OqFfea0dXdd9vqai=hGuQ8kuc9pgc9s8qqaq=dirpe0xb9q8qiLsFr0=vr0=vr0dc8meaabaqaciaacaGaaeqabaqabeGadaaakeaacqWG0baDcqqGjbqscqqGebarcqqGsbGucqGGOaakiiGacqWFZoWzcqGGPaqkcqGH9aqpdaWcaaqaaiabcocaJiabbsha0jabbkhaYjabbwha1jabbwgaLjabbccaGiabbEgaNjabbwgaLjabb6gaUjabbwgaLjabbohaZjabbccaGiabcUfaBjabcIcaOiabdseaenaaBaaaleaacqWGNbWzaeqaaOGaeyyzImRae83SdCMaeiykaKIaeeiiaaIaeeyAaKMaeeOBa4MaeeiiaaIaeeyBa0MaeeyzauMaeeiDaqNaeeyyaeMaeeyla0IaeeyyaeMaeeOBa4MaeeyyaeMaeeiBaWMaeeyEaKNaee4CamNaeeyAaKMaee4CamNaeiyxa0LaeeiiaaIaeeyyaeMaeeOBa4MaeeizaqMaeeiiaaIaei4waSLaeiikaGIaemiraq0aaSbaaSqaaiabdEgaNbqabaGccqGH8aapcqWFZoWzcqGGPaqkcqqGGaaicqqGPbqAcqqGUbGBcqqGGaaicqqGHbqycqqGSbaBcqqGSbaBcqqGGaaicqqGPbqAcqqGUbGBcqqGKbazcqqGPbqAcqqG2bGDcqqGPbqAcqqGKbazcqqG1bqDcqqGHbqycqqGSbaBcqqGGaaicqqGZbWCcqqG0baDcqqG1bqDcqqGKbazcqqGPbqAcqqGLbqzcqqGZbWCcqGGDbqxaeaacqGGJaWicqqG0baDcqqGYbGCcqqG1bqDcqqGLbqzcqqGGaaicqqGNbWzcqqGLbqzcqqGUbGBcqqGLbqzcqqGZbWCcqqGGaaicqGGBbWwcqGGOaakcqWGebardaWgaaWcbaGaem4zaCgabeaakiabgwMiZkab=n7aNjabcMcaPiabbccaGiabbMgaPjabb6gaUjabbccaGiabb2gaTjabbwgaLjabbsha0jabbggaHjabb2caTiabbggaHjabb6gaUjabbggaHjabbYgaSjabbMha5jabbohaZjabbMgaPjabbohaZjabb2faDbaacqGGUaGlaaa@C40A@

Stevens and Doerge [3] define the integration-driven revision rate (IRR) as the percent of genes that are declared differentially expressed in individual studies but not in meta-analysis. IRR represents the genes that are missed or "dropped" in meta-analysis versus separate study analyses:

IRR(γ)=#genes [(Dg≥γ) in at least one individual study] and [(Dg<γ) in meta-analysis]#genes [(Dg≥γ) in at least one individual study].
 MathType@MTEF@5@5@+=feaafiart1ev1aaatCvAUfKttLearuWrP9MDH5MBPbIqV92AaeXatLxBI9gBaebbnrfifHhDYfgasaacH8akY=wiFfYdH8Gipec8Eeeu0xXdbba9frFj0=OqFfea0dXdd9vqai=hGuQ8kuc9pgc9s8qqaq=dirpe0xb9q8qiLsFr0=vr0=vr0dc8meaabaqaciaacaGaaeqabaqabeGadaaakeaacqqGjbqscqqGsbGucqqGsbGucqGGOaakiiGacqWFZoWzcqGGPaqkcqGH9aqpdaWcaaqaaiabcocaJiabbEgaNjabbwgaLjabb6gaUjabbwgaLjabbohaZjabbccaGiabcUfaBjabcIcaOiabdseaenaaBaaaleaacqWGNbWzaeqaaOGaeyyzImRae83SdCMaeiykaKIaeeiiaaIaeeyAaKMaeeOBa4MaeeiiaaIaeeyyaeMaeeiDaqNaeeiiaaIaeeiBaWMaeeyzauMaeeyyaeMaee4CamNaeeiDaqNaeeiiaaIaee4Ba8MaeeOBa4MaeeyzauMaeeiiaaIaeeyAaKMaeeOBa4MaeeizaqMaeeyAaKMaeeODayNaeeyAaKMaeeizaqMaeeyDauNaeeyyaeMaeeiBaWMaeeiiaaIaee4CamNaeeiDaqNaeeyDauNaeeizaqMaeeyEaKNaeiyxa0LaeeiiaaIaeeyyaeMaeeOBa4MaeeizaqMaeeiiaaIaei4waSLaeiikaGIaemiraq0aaSbaaSqaaiabdEgaNbqabaGccqGH8aapcqWFZoWzcqGGPaqkcqqGGaaicqqGPbqAcqqGUbGBcqqGGaaicqqGTbqBcqqGLbqzcqqG0baDcqqGHbqycqqGTaqlcqqGHbqycqqGUbGBcqqGHbqycqqGSbaBcqqG5bqEcqqGZbWCcqqGPbqAcqqGZbWCcqGGDbqxaeaacqGGJaWicqqGNbWzcqqGLbqzcqqGUbGBcqqGLbqzcqqGZbWCcqqGGaaicqGGBbWwcqGGOaakcqWGebardaWgaaWcbaGaem4zaCgabeaakiabgwMiZkab=n7aNjabcMcaPiabbccaGiabbMgaPjabb6gaUjabbccaGiabbggaHjabbsha0jabbccaGiabbYgaSjabbwgaLjabbggaHjabbohaZjabbsha0jabbccaGiabb+gaVjabb6gaUjabbwgaLjabbccaGiabbMgaPjabb6gaUjabbsgaKjabbMgaPjabbAha2jabbMgaPjabbsgaKjabbwha1jabbggaHjabbYgaSjabbccaGiabbohaZjabbsha0jabbwha1jabbsgaKjabbMha5jabb2faDbaacqGGUaGlaaa@D2A2@

The corresponding true integration-driven revision rate, *t*IRR, is the percent of true genes that are identified as differentially expressed in at least one individual study but not in meta-analysis:

tIRR(γ)=#true genes [(Dg≥γ) in at least one individual study] and [(Dg<γ) in meta-analysis]#true genes [(Dg≥γ) in at least one individual study].
 MathType@MTEF@5@5@+=feaafiart1ev1aaatCvAUfKttLearuWrP9MDH5MBPbIqV92AaeXatLxBI9gBaebbnrfifHhDYfgasaacH8akY=wiFfYdH8Gipec8Eeeu0xXdbba9frFj0=OqFfea0dXdd9vqai=hGuQ8kuc9pgc9s8qqaq=dirpe0xb9q8qiLsFr0=vr0=vr0dc8meaabaqaciaacaGaaeqabaqabeGadaaakeaacqWG0baDcqqGjbqscqqGsbGucqqGsbGucqGGOaakiiGacqWFZoWzcqGGPaqkcqGH9aqpdaWcaaqaaiabcocaJiabbsha0jabbkhaYjabbwha1jabbwgaLjabbccaGiabbEgaNjabbwgaLjabb6gaUjabbwgaLjabbohaZjabbccaGiabcUfaBjabcIcaOiabdseaenaaBaaaleaacqWGNbWzaeqaaOGaeyyzImRae83SdCMaeiykaKIaeeiiaaIaeeyAaKMaeeOBa4MaeeiiaaIaeeyyaeMaeeiDaqNaeeiiaaIaeeiBaWMaeeyzauMaeeyyaeMaee4CamNaeeiDaqNaeeiiaaIaee4Ba8MaeeOBa4MaeeyzauMaeeiiaaIaeeyAaKMaeeOBa4MaeeizaqMaeeyAaKMaeeODayNaeeyAaKMaeeizaqMaeeyDauNaeeyyaeMaeeiBaWMaeeiiaaIaee4CamNaeeiDaqNaeeyDauNaeeizaqMaeeyEaKNaeiyxa0LaeeiiaaIaeeyyaeMaeeOBa4MaeeizaqMaeeiiaaIaei4waSLaeiikaGIaemiraq0aaSbaaSqaaiabdEgaNbqabaGccqGH8aapcqWFZoWzcqGGPaqkcqqGGaaicqqGPbqAcqqGUbGBcqqGGaaicqqGTbqBcqqGLbqzcqqG0baDcqqGHbqycqqGTaqlcqqGHbqycqqGUbGBcqqGHbqycqqGSbaBcqqG5bqEcqqGZbWCcqqGPbqAcqqGZbWCcqGGDbqxaeaacqGGJaWicqqG0baDcqqGYbGCcqqG1bqDcqqGLbqzcqqGGaaicqqGNbWzcqqGLbqzcqqGUbGBcqqGLbqzcqqGZbWCcqqGGaaicqGGBbWwcqGGOaakcqWGebardaWgaaWcbaGaem4zaCgabeaakiabgwMiZkab=n7aNjabcMcaPiabbccaGiabbMgaPjabb6gaUjabbccaGiabbggaHjabbsha0jabbccaGiabbYgaSjabbwgaLjabbggaHjabbohaZjabbsha0jabbccaGiabb+gaVjabb6gaUjabbwgaLjabbccaGiabbMgaPjabb6gaUjabbsgaKjabbMgaPjabbAha2jabbMgaPjabbsgaKjabbwha1jabbggaHjabbYgaSjabbccaGiabbohaZjabbsha0jabbwha1jabbsgaKjabbMha5jabb2faDbaacqGGUaGlaaa@E0D9@

### Bayesian false discovery rate

The false discovery rate (FDR) was introduced by Benjamini and Hochberg [52], and is defined as the expected number of discovered genes that are not truly differentially expressed divided by the total number of discovered genes. Further discussion and application of FDR in a microarray context include Tusher *et al*. [53], Storey [54], Storey and Tibshirani [55] and Genovese and Wasserman [56]. In a Bayesian approach, Genovese and Wasserman [57] defined the posterior expected FDR (*pe*FDR) as:

peFDR=E(FDR|Y)=∑g(1−Dg)δg∑gδg,
 MathType@MTEF@5@5@+=feaafiart1ev1aaatCvAUfKttLearuWrP9MDH5MBPbIqV92AaeXatLxBI9gBaebbnrfifHhDYfgasaacH8akY=wiFfYdH8Gipec8Eeeu0xXdbba9frFj0=OqFfea0dXdd9vqai=hGuQ8kuc9pgc9s8qqaq=dirpe0xb9q8qiLsFr0=vr0=vr0dc8meaabaqaciaacaGaaeqabaqabeGadaaakeaacqWGWbaCcqWGLbqzcqqGgbGrcqqGebarcqqGsbGucqGH9aqpcqWGfbqrcqGGOaakcqqGgbGrcqqGebarcqqGsbGucqGG8baFieWacqWFzbqwcqGGPaqkcqGH9aqpdaWcaaqaamaaqafabaGaeiikaGIaeGymaeJaeyOeI0Iaemiraq0aaSbaaSqaaiabdEgaNbqabaGccqGGPaqkiiGacqGF0oazdaWgaaWcbaGaem4zaCgabeaaaeaacqWGNbWzaeqaniabggHiLdaakeaadaaeqbqaaiab+r7aKnaaBaaaleaacqWGNbWzaeqaaaqaaiabdEgaNbqab0GaeyyeIuoaaaGccqGGSaalaaa@51F5@

where *δ*_*g *_is an indicator for differential expression and ***Y ***represents the data (see also Do *et al*. [28]).

### Two-study simulation results

We simulated data for two studies, Study 1 and Study 2, with 3,000 genes and a format similar to the *B. subtilis *mutant and induction studies. We used three levels for the percent of differentially expressed genes (denoted *p*_*s *_to indicate *simulated), p*_*s *_= 5%, 10%, 25%, and three mean levels of inter-study variation τg2
 MathType@MTEF@5@5@+=feaafiart1ev1aaatCvAUfKttLearuWrP9MDH5MBPbIqV92AaeXatLxBI9gBaebbnrfifHhDYfgasaacH8akY=wiFfYdH8Gipec8Eeeu0xXdbba9frFj0=OqFfea0dXdd9vqai=hGuQ8kuc9pgc9s8qqaq=dirpe0xb9q8qiLsFr0=vr0=vr0dc8meaabaqaciaacaGaaeqabaqabeGadaaakeaaiiGacqWFepaDdaqhaaWcbaGaem4zaCgabaGaeGOmaidaaaaa@30EE@. We refer to the mean levels of inter-study variability as low, medium and high, based on comparison to the biological data, as follows. Each level of τg2
 MathType@MTEF@5@5@+=feaafiart1ev1aaatCvAUfKttLearuWrP9MDH5MBPbIqV92AaeXatLxBI9gBaebbnrfifHhDYfgasaacH8akY=wiFfYdH8Gipec8Eeeu0xXdbba9frFj0=OqFfea0dXdd9vqai=hGuQ8kuc9pgc9s8qqaq=dirpe0xb9q8qiLsFr0=vr0=vr0dc8meaabaqaciaacaGaaeqabaqabeGadaaakeaaiiGacqWFepaDdaqhaaWcbaGaem4zaCgabaGaeGOmaidaaaaa@30EE@ was simulated from a Normal distribution with the following means: low variability: mean τg2
 MathType@MTEF@5@5@+=feaafiart1ev1aaatCvAUfKttLearuWrP9MDH5MBPbIqV92AaeXatLxBI9gBaebbnrfifHhDYfgasaacH8akY=wiFfYdH8Gipec8Eeeu0xXdbba9frFj0=OqFfea0dXdd9vqai=hGuQ8kuc9pgc9s8qqaq=dirpe0xb9q8qiLsFr0=vr0=vr0dc8meaabaqaciaacaGaaeqabaqabeGadaaakeaaiiGacqWFepaDdaqhaaWcbaGaem4zaCgabaGaeGOmaidaaaaa@30EE@ = 0.1 for differentially expressed and mean τg2
 MathType@MTEF@5@5@+=feaafiart1ev1aaatCvAUfKttLearuWrP9MDH5MBPbIqV92AaeXatLxBI9gBaebbnrfifHhDYfgasaacH8akY=wiFfYdH8Gipec8Eeeu0xXdbba9frFj0=OqFfea0dXdd9vqai=hGuQ8kuc9pgc9s8qqaq=dirpe0xb9q8qiLsFr0=vr0=vr0dc8meaabaqaciaacaGaaeqabaqabeGadaaakeaaiiGacqWFepaDdaqhaaWcbaGaem4zaCgabaGaeGOmaidaaaaa@30EE@ = 0.01 for non-expressed genes; medium: mean τg2
 MathType@MTEF@5@5@+=feaafiart1ev1aaatCvAUfKttLearuWrP9MDH5MBPbIqV92AaeXatLxBI9gBaebbnrfifHhDYfgasaacH8akY=wiFfYdH8Gipec8Eeeu0xXdbba9frFj0=OqFfea0dXdd9vqai=hGuQ8kuc9pgc9s8qqaq=dirpe0xb9q8qiLsFr0=vr0=vr0dc8meaabaqaciaacaGaaeqabaqabeGadaaakeaaiiGacqWFepaDdaqhaaWcbaGaem4zaCgabaGaeGOmaidaaaaa@30EE@ = 0.3 and 0.03; and high: mean τg2
 MathType@MTEF@5@5@+=feaafiart1ev1aaatCvAUfKttLearuWrP9MDH5MBPbIqV92AaeXatLxBI9gBaebbnrfifHhDYfgasaacH8akY=wiFfYdH8Gipec8Eeeu0xXdbba9frFj0=OqFfea0dXdd9vqai=hGuQ8kuc9pgc9s8qqaq=dirpe0xb9q8qiLsFr0=vr0=vr0dc8meaabaqaciaacaGaaeqabaqabeGadaaakeaaiiGacqWFepaDdaqhaaWcbaGaem4zaCgabaGaeGOmaidaaaaa@30EE@ = 0.7 and 0.07, for expressed and non-expressed genes, respectively. The variances of the Normal distributions were equivalent to the biological data (for more detail, see Methods). Again, we refer to genes that were simulated to be differentially expressed as true genes. Each array was standardized to have mean zero and unit standard deviation.

#### Bayesian standardized expression integration model: modeling inter-study variation

For the Bayesian standardized expression integration meta-analysis model, the prior distribution assigned to τg2
 MathType@MTEF@5@5@+=feaafiart1ev1aaatCvAUfKttLearuWrP9MDH5MBPbIqV92AaeXatLxBI9gBaebbnrfifHhDYfgasaacH8akY=wiFfYdH8Gipec8Eeeu0xXdbba9frFj0=OqFfea0dXdd9vqai=hGuQ8kuc9pgc9s8qqaq=dirpe0xb9q8qiLsFr0=vr0=vr0dc8meaabaqaciaacaGaaeqabaqabeGadaaakeaaiiGacqWFepaDdaqhaaWcbaGaem4zaCgabaGaeGOmaidaaaaa@30EE@ has a large influence on the results. We considered three prior distributions with extensive use in hierarchical Bayesian meta-analytic models: inverse-gamma prior distributions (see, for example, Choi *et al*. [2], Smith *et al*. [34], Normand [35], Sargent *et al*. [38]), log-logistic (DuMouchel and Normand [36]), and locally uniform (Gelman *et al*. [39]). See Methods for a detailed description of these prior assignments. Briefly, the informative inverse-gamma and locally uniform distributions pooled information from sets of genes to provide better estimates of inter-study variability. The log-logistic prior distribution treated each gene separately without pooling information from sets of genes, and was a function of the weighted average of the sampling variabilities across studies for each gene. The log-logistic distribution prevents the meta-analysis results from being overly influenced by studies with large sampling variability. Of the prior specifications, only the locally uniform prior improved upon individual study analyses. In particular, this prior distribution was centered at the median of the inter-study variability based on the data, with separate distributions for the differentially expressed and non-expressed genes (see Methods). We show results hereafter based on this prior distribution.

#### Standardized expression integration versus probability integration model

**Figure 1:**
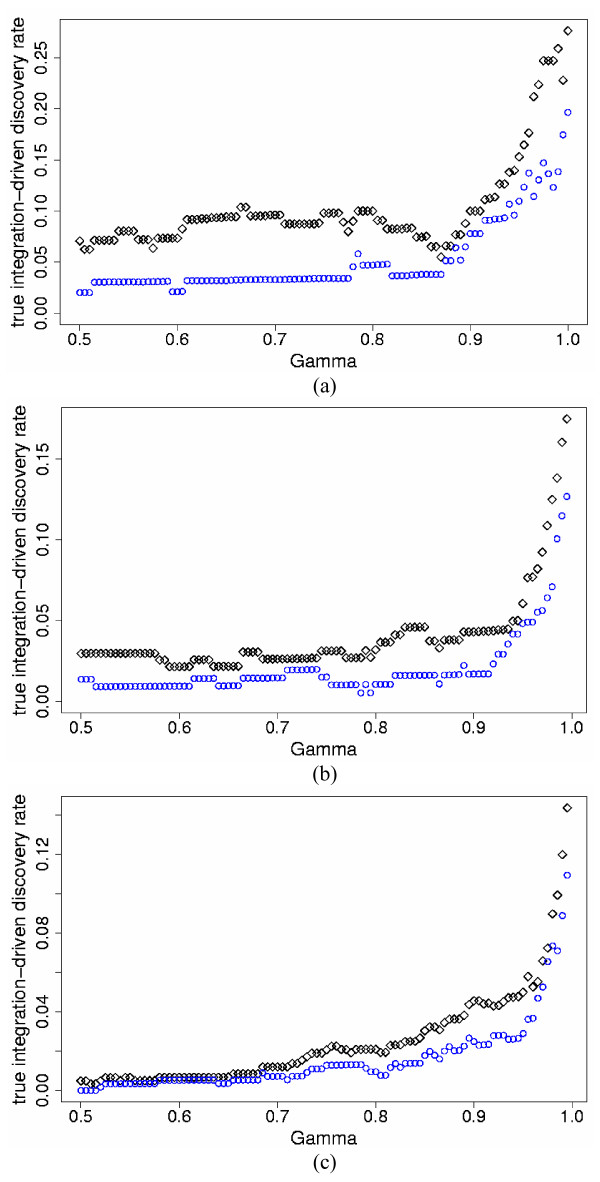
***t*IDR versus posterior probability of differential expression for the two-study simulation data**. True integration-driven discovery rate (*t*IDR) versus threshold values of posterior probability of differential expression *γ *≥ 0.50, for the standardized expression integration model (blue circles) and probability integration model (black diamonds) for the two-study simulation data with high mean τg2
 MathType@MTEF@5@5@+=feaafiart1ev1aaatCvAUfKttLearuWrP9MDH5MBPbIqV92AaeXatLxBI9gBaebbnrfifHhDYfgasaacH8akY=wiFfYdH8Gipec8Eeeu0xXdbba9frFj0=OqFfea0dXdd9vqai=hGuQ8kuc9pgc9s8qqaq=dirpe0xb9q8qiLsFr0=vr0=vr0dc8meaabaqaciaacaGaaeqabaqabeGadaaakeaaiiGacqWFepaDdaqhaaWcbaGaem4zaCgabaGaeGOmaidaaaaa@30EE@ = 0.7 (differentially expressed); 0.07 (non-differentially expressed) and the following simulated percent differentially expressed genes *p*_*s*_: a) 5%; b) 10%; c) 25%.

**Figure 2:**
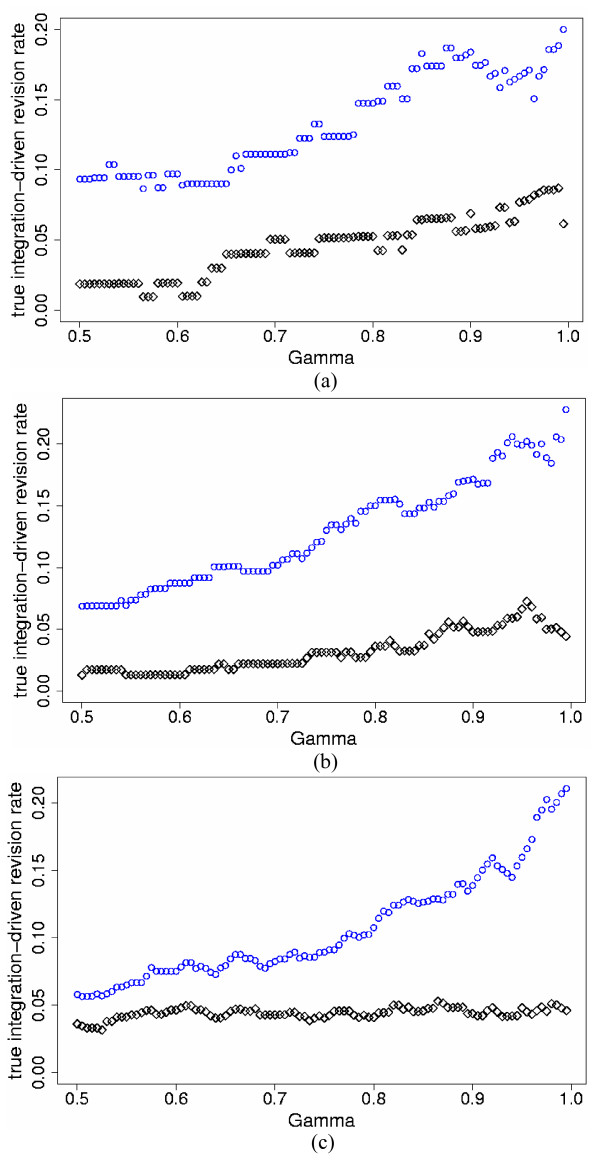
***t*IRR versus posterior probability of differential expression for the two-study simulation data**. True integration-driven revision rate (*t*IRR) versus threshold values of posterior probability of differential expression *γ *≥ 0.50, for the standardized expression integration model (blue circles) and probability integration model (black diamonds) for the two-study simulation data with high mean τg2
 MathType@MTEF@5@5@+=feaafiart1ev1aaatCvAUfKttLearuWrP9MDH5MBPbIqV92AaeXatLxBI9gBaebbnrfifHhDYfgasaacH8akY=wiFfYdH8Gipec8Eeeu0xXdbba9frFj0=OqFfea0dXdd9vqai=hGuQ8kuc9pgc9s8qqaq=dirpe0xb9q8qiLsFr0=vr0=vr0dc8meaabaqaciaacaGaaeqabaqabeGadaaakeaaiiGacqWFepaDdaqhaaWcbaGaem4zaCgabaGaeGOmaidaaaaa@30EE@ = 0.7 (differentially expressed); 0.07 (non-differentially expressed) and the following simulated percent differentially expressed genes *p*_*s*_: a) 5%; b) 10%; c) 25%.

We implemented the Bayesian standardized expression integration model (SEI hereafter, Model (1)) and the Bayesian probability integration model (PI hereafter, Model (2)) to combine the two simulated studies for the three levels of percent differentially expressed genes and three levels of inter-study variation. We also analyzed each study individually. Note again that in individual studies, the SEI and PI models are equivalent, i.e. the only differences between the SEI and PI models are at the inter-study level. In order to compare the SEI and PI models, we calculated for each model the true integration-driven discovery rate (*t*IDR) and the true integration-driven revision rate (*t*IRR) for thresholds of *γ *≥ 0.50, i.e. the posterior probability of differential expression greater or equal to 50%. The PI model produced higher *t*IDR and lower *t*IRR than the SEI model for all values of *γ *≥ 0.50 for the simulated data. Figures 1 and 2 display the *t*IDR and *t*IRR results, respectively, for the three simulated levels of *p*_*s *_and high mean τg2
 MathType@MTEF@5@5@+=feaafiart1ev1aaatCvAUfKttLearuWrP9MDH5MBPbIqV92AaeXatLxBI9gBaebbnrfifHhDYfgasaacH8akY=wiFfYdH8Gipec8Eeeu0xXdbba9frFj0=OqFfea0dXdd9vqai=hGuQ8kuc9pgc9s8qqaq=dirpe0xb9q8qiLsFr0=vr0=vr0dc8meaabaqaciaacaGaaeqabaqabeGadaaakeaaiiGacqWFepaDdaqhaaWcbaGaem4zaCgabaGaeGOmaidaaaaa@30EE@. Table 1 presents results for all simulated data sets, for representative threshold value *γ *= 0.95.

**Table 1:** Results for two-study simulation data. True integration-driven discovery rate (*t*IDR) and true integration-driven revision rate (*t*IRR) for threshold value of posterior probability of differential expression *γ *= 0.95, and the number of true discovered genes for posterior expected false discovery rate *pe*FDR = 5%, for the Bayesian standardized expression integration (SEI) and probability integration (PI) models. Results are shown for the three levels of simulated percent differentially expressed genes *p*_*s *_and three levels of mean inter-study variability τg2
 MathType@MTEF@5@5@+=feaafiart1ev1aaatCvAUfKttLearuWrP9MDH5MBPbIqV92AaeXatLxBI9gBaebbnrfifHhDYfgasaacH8akY=wiFfYdH8Gipec8Eeeu0xXdbba9frFj0=OqFfea0dXdd9vqai=hGuQ8kuc9pgc9s8qqaq=dirpe0xb9q8qiLsFr0=vr0=vr0dc8meaabaqaciaacaGaaeqabaqabeGadaaakeaaiiGacqWFepaDdaqhaaWcbaGaem4zaCgabaGaeGOmaidaaaaa@30EE@ for the two-study simulation data.

		***p*_*s *_= 5%**		***p*_*s *_= 10%**		***p*_*s *_= 25%**	
		
**Two-Study Simulation Data**		**SEI**	**PI**	**SEI**	**PI**	**SEI**	**PI**
**Low: mean **τg2 MathType@MTEF@5@5@+=feaafiart1ev1aaatCvAUfKttLearuWrP9MDH5MBPbIqV92AaeXatLxBI9gBaebbnrfifHhDYfgasaacH8akY=wiFfYdH8Gipec8Eeeu0xXdbba9frFj0=OqFfea0dXdd9vqai=hGuQ8kuc9pgc9s8qqaq=dirpe0xb9q8qiLsFr0=vr0=vr0dc8meaabaqaciaacaGaaeqabaqabeGadaaakeaaiiGacqWFepaDdaqhaaWcbaGaem4zaCgabaGaeGOmaidaaaaa@30EE@**= 0.1 (DE); 0.01 (non DE)**	***t*IDR, *γ *= 0.95**	24.6%	29.9%	12.9%	18.9%	3.8%	9.0%
	***t*IRR, *γ *= 0.95**	3.7%	0%	2.9%	1.4%	6.2%	1.5%
	**True Genes, *pe*FDR = 5%**	84	92	183	196	479	513

**Medium: mean **τg2 MathType@MTEF@5@5@+=feaafiart1ev1aaatCvAUfKttLearuWrP9MDH5MBPbIqV92AaeXatLxBI9gBaebbnrfifHhDYfgasaacH8akY=wiFfYdH8Gipec8Eeeu0xXdbba9frFj0=OqFfea0dXdd9vqai=hGuQ8kuc9pgc9s8qqaq=dirpe0xb9q8qiLsFr0=vr0=vr0dc8meaabaqaciaacaGaaeqabaqabeGadaaakeaaiiGacqWFepaDdaqhaaWcbaGaem4zaCgabaGaeGOmaidaaaaa@30EE@**= 0.3 (DE); 0.03 (non DE)**	***t*IDR, *γ *= 0.95**	11.2%	19.5%	10.8%	16.2%	2.6%	6.4%
	***t*IRR, *γ *= 0.95**	16.4%	7.5%	15.9%	4.5%	16.4%	3.1%
	**True Genes, *pe*FDR = 5%**	80	97	185	211	493	547

**High: mean **τg2 MathType@MTEF@5@5@+=feaafiart1ev1aaatCvAUfKttLearuWrP9MDH5MBPbIqV92AaeXatLxBI9gBaebbnrfifHhDYfgasaacH8akY=wiFfYdH8Gipec8Eeeu0xXdbba9frFj0=OqFfea0dXdd9vqai=hGuQ8kuc9pgc9s8qqaq=dirpe0xb9q8qiLsFr0=vr0=vr0dc8meaabaqaciaacaGaaeqabaqabeGadaaakeaaiiGacqWFepaDdaqhaaWcbaGaem4zaCgabaGaeGOmaidaaaaa@30EE@**= 0.7(DE); 0.07(non DE)**	***t*IDR, *γ *= 0.95**	11.0%	15.3%	5.4%	7.6%	2.9%	4.9%
	***t*IRR, *γ *= 0.95**	16.7%	7.7%	19.9%	6.6%	16.0%	4.8%
	**True Genes, *pe*FDR = 5%**	93	105	212	235	572	617

**Figure 3:**
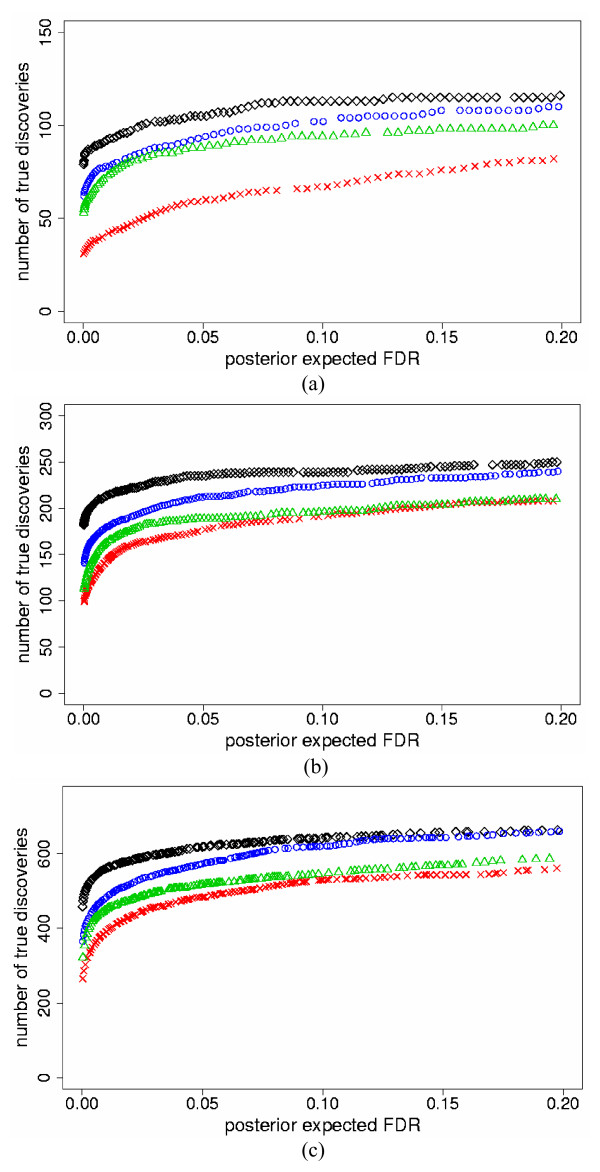
**Number of true discovered genes versus *pe*FDR for the two-study simulation data**. The maximum number of true discovered genes versus posterior expected false discovery rate (*pe*FDR) for the standardized expression integration model (blue circles), probability integration model (black diamonds), individual analyses of Study 1 (red checks) and Study 2 (green triangles), for the two-study simulation data with high mean τg2
 MathType@MTEF@5@5@+=feaafiart1ev1aaatCvAUfKttLearuWrP9MDH5MBPbIqV92AaeXatLxBI9gBaebbnrfifHhDYfgasaacH8akY=wiFfYdH8Gipec8Eeeu0xXdbba9frFj0=OqFfea0dXdd9vqai=hGuQ8kuc9pgc9s8qqaq=dirpe0xb9q8qiLsFr0=vr0=vr0dc8meaabaqaciaacaGaaeqabaqabeGadaaakeaaiiGacqWFepaDdaqhaaWcbaGaem4zaCgabaGaeGOmaidaaaaa@30EE@ = 0.7 (differentially expressed); 0.07 (non-differentially expressed) and the following simulated percent differentially expressed genes *p*_*s*_: a) 5%; b) 10%; c) 25%.

We also fixed levels of *pe*FDR and compared the number of true discoveries for the two models. We found that both models improved the number of true discoveries versus individual analyses, and the PI model identified more true genes than the SEI model for the same levels of *pe*FDR < 20%. Figure 3 illustrates the results for the three *p*_*s *_levels and high mean τg2
 MathType@MTEF@5@5@+=feaafiart1ev1aaatCvAUfKttLearuWrP9MDH5MBPbIqV92AaeXatLxBI9gBaebbnrfifHhDYfgasaacH8akY=wiFfYdH8Gipec8Eeeu0xXdbba9frFj0=OqFfea0dXdd9vqai=hGuQ8kuc9pgc9s8qqaq=dirpe0xb9q8qiLsFr0=vr0=vr0dc8meaabaqaciaacaGaaeqabaqabeGadaaakeaaiiGacqWFepaDdaqhaaWcbaGaem4zaCgabaGaeGOmaidaaaaa@30EE@. Table 1 reports results for all simulations, for representative *pe*FDR = 5%.

The primary difference between the two models is that the SEI model first combines the mean standardized gene expression levels from each study into an overall mean and then calculates the probability of differential expression based on this overall mean, while the PI model calculates the probability of differential expression based on the separate study means. For genes that have high mean standardized expression in one study but lower mean standardized expression in a second study, the SEI model tends to identify these genes as non-differentially expressed based on an approximately medium overall mean; however, the PI model identifies more of these types of genes as differentially expressed. This is due to keeping the study means separate in the PI model, and thus genes with high probability of differential expression in one study are not overly offset by genes with lower probability of differential expression in the second study. Due to these model differences, the SEI model declares more true genes as non-differentially expressed, thus producing higher *t*IRR than the PI model. For genes that are declared non-differentially expressed in both individual studies, the PI model identifies more of these genes as differentially expressed in meta-analysis versus the SEI model, resulting in higher *t*IDR and more true genes identified for the same level of false discovery than the SEI model.

### Five-study simulation results

**Figure 4:**
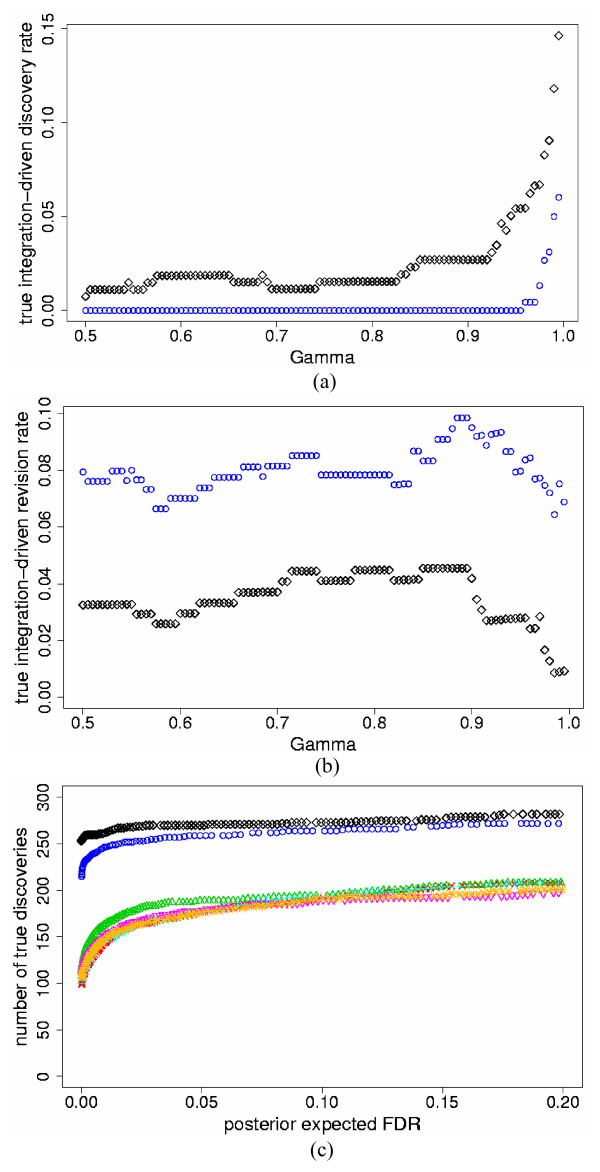
***t*IDR, *t*IRR and true discovered genes versus *pe*FDR for the five-study simulation data**. a) True integration-driven discovery rate (*t*IDR) versus threshold values of posterior probability of differential expression *γ *≥ 0.50, for the standardized expression integration model (blue circles) and probability integration model (black diamonds) for the five-study simulation data with high mean τg2
 MathType@MTEF@5@5@+=feaafiart1ev1aaatCvAUfKttLearuWrP9MDH5MBPbIqV92AaeXatLxBI9gBaebbnrfifHhDYfgasaacH8akY=wiFfYdH8Gipec8Eeeu0xXdbba9frFj0=OqFfea0dXdd9vqai=hGuQ8kuc9pgc9s8qqaq=dirpe0xb9q8qiLsFr0=vr0=vr0dc8meaabaqaciaacaGaaeqabaqabeGadaaakeaaiiGacqWFepaDdaqhaaWcbaGaem4zaCgabaGaeGOmaidaaaaa@30EE@ = 0.7 (differentially expressed); 0.07 (non-differentially expressed) and simulated percent differentially expressed genes *p*_*s *_= 10%; b) True integration-driven revision rate (*t*IRR) versus threshold values of posterior probability of differential expression *γ *≥ 0.50, for the standardized expression integration model (blue circles) and probability integration model (black diamonds) for the five-study simulation data with high mean τg2
 MathType@MTEF@5@5@+=feaafiart1ev1aaatCvAUfKttLearuWrP9MDH5MBPbIqV92AaeXatLxBI9gBaebbnrfifHhDYfgasaacH8akY=wiFfYdH8Gipec8Eeeu0xXdbba9frFj0=OqFfea0dXdd9vqai=hGuQ8kuc9pgc9s8qqaq=dirpe0xb9q8qiLsFr0=vr0=vr0dc8meaabaqaciaacaGaaeqabaqabeGadaaakeaaiiGacqWFepaDdaqhaaWcbaGaem4zaCgabaGaeGOmaidaaaaa@30EE@ = 0.7 (differentially expressed); 0.07 (non-differentially expressed) and simulated percent differentially expressed genes *p*_*s *_= 10%; c) The maximum number of true discovered genes versus posterior expected false discovery rate (*pe*FDR) for the standardized expression integration model (blue circles), probability integration model (black diamonds), individual analyses of Study 1 (red checks), Study 2 (green triangles), Study 3 (turquoise pluses), Study 4 (pink inverted triangles), Study 5 (gold stars) for the five-study simulation data with high mean τg2
 MathType@MTEF@5@5@+=feaafiart1ev1aaatCvAUfKttLearuWrP9MDH5MBPbIqV92AaeXatLxBI9gBaebbnrfifHhDYfgasaacH8akY=wiFfYdH8Gipec8Eeeu0xXdbba9frFj0=OqFfea0dXdd9vqai=hGuQ8kuc9pgc9s8qqaq=dirpe0xb9q8qiLsFr0=vr0=vr0dc8meaabaqaciaacaGaaeqabaqabeGadaaakeaaiiGacqWFepaDdaqhaaWcbaGaem4zaCgabaGaeGOmaidaaaaa@30EE@ = 0.7 (differentially expressed); 0.07 (non-differentially expressed) and simulated percent differentially expressed genes *p*_*s *_= 10%.

We also compared the SEI and PI models for five independent studies. For this, we simulated three additional studies with format similar to Study 1, but with different parameter values for slide and experiment variation (see Methods). All other parameter specifications were similar to the two-study simulations. In all data sets, the PI model again identified higher *t*IDR and lower *t*IRR than the SEI model for all thresholds of *γ *≥ 0.50. Figures 4a and 4b show results for *p*_*s *_= 10% and high mean τg2
 MathType@MTEF@5@5@+=feaafiart1ev1aaatCvAUfKttLearuWrP9MDH5MBPbIqV92AaeXatLxBI9gBaebbnrfifHhDYfgasaacH8akY=wiFfYdH8Gipec8Eeeu0xXdbba9frFj0=OqFfea0dXdd9vqai=hGuQ8kuc9pgc9s8qqaq=dirpe0xb9q8qiLsFr0=vr0=vr0dc8meaabaqaciaacaGaaeqabaqabeGadaaakeaaiiGacqWFepaDdaqhaaWcbaGaem4zaCgabaGaeGOmaidaaaaa@30EE@; Table 2 details the results for all data sets, with representative threshold value *γ *= 0.95. Note that the SEI model produced *t*IDR = 0% for many levels of *γ*. In these incidents, for all true genes identified by the SEI model, at least one of the five individual studies had *D*_*g *_at least as high as *γ*. When compared to the two-study simulations, combining more studies resulted in lower *t*IDR in most cases for both the SEI and PI models. This occurred since, for a larger number of studies, it was more likely that some genes had *D*_*g *_≥ *γ *in at least one individual study, which reduced *t*IDR. The *t*IRR was also lower in most cases in comparison to the two-study simulations for both the SEI and PI models. This was due to the increase in *D*_*g *_in the meta-analysis models when combining more studies, which reduced *t*IRR.

**Table 2:** Results for five-study simulation data. True integration-driven discovery rate (*t*IDR) and true integration-driven revision rate (*t*IRR) for threshold value of posterior probability of differential expression *γ *= 0.95, and the number of true discovered genes for posterior expected false discovery rate *pe*FDR = 5%, for the Bayesian standardized expression integration (SEI) and probability integration (PI) models. Results are shown for the three levels of simulated percent differentially expressed genes *p*_*s *_and three levels of mean inter-study variability τg2
 MathType@MTEF@5@5@+=feaafiart1ev1aaatCvAUfKttLearuWrP9MDH5MBPbIqV92AaeXatLxBI9gBaebbnrfifHhDYfgasaacH8akY=wiFfYdH8Gipec8Eeeu0xXdbba9frFj0=OqFfea0dXdd9vqai=hGuQ8kuc9pgc9s8qqaq=dirpe0xb9q8qiLsFr0=vr0=vr0dc8meaabaqaciaacaGaaeqabaqabeGadaaakeaaiiGacqWFepaDdaqhaaWcbaGaem4zaCgabaGaeGOmaidaaaaa@30EE@ for the five-study simulation data.

		***p*_*s *_= 5%**		***p*_*s *_= 10%**		***p*_*s *_= 25%**	
		
**Five-Study Simulation Data**		**SEI**	**PI**	**SEI**	**PI**	**SEI**	**PI**
**Low : mean **τg2 MathType@MTEF@5@5@+=feaafiart1ev1aaatCvAUfKttLearuWrP9MDH5MBPbIqV92AaeXatLxBI9gBaebbnrfifHhDYfgasaacH8akY=wiFfYdH8Gipec8Eeeu0xXdbba9frFj0=OqFfea0dXdd9vqai=hGuQ8kuc9pgc9s8qqaq=dirpe0xb9q8qiLsFr0=vr0=vr0dc8meaabaqaciaacaGaaeqabaqabeGadaaakeaaiiGacqWFepaDdaqhaaWcbaGaem4zaCgabaGaeGOmaidaaaaa@30EE@**= 0.1 (DE); 0.01(non DE)**	***t*IRR, *γ *= 0.95**	13.7%	34.7%	3.8%	20.6%	0%	11.4%
	***t*IRR, *γ *= 0.95**	4.5%	0%	6.7%	0.6%	15.4%	1.4%
	**True Genes, *pe*FDR = 5%**	88	106	187	215	496	576

**Medium : mean **τg2 MathType@MTEF@5@5@+=feaafiart1ev1aaatCvAUfKttLearuWrP9MDH5MBPbIqV92AaeXatLxBI9gBaebbnrfifHhDYfgasaacH8akY=wiFfYdH8Gipec8Eeeu0xXdbba9frFj0=OqFfea0dXdd9vqai=hGuQ8kuc9pgc9s8qqaq=dirpe0xb9q8qiLsFr0=vr0=vr0dc8meaabaqaciaacaGaaeqabaqabeGadaaakeaaiiGacqWFepaDdaqhaaWcbaGaem4zaCgabaGaeGOmaidaaaaa@30EE@**= 0.3 (DE); 0.03 (non DE)**	***t*IDR, *γ *= 0.95**	7.2%	21.3%	2.2%	15.2%	0%	6.7%
	***t*IRR, *γ *= 0.95**	9.4%	0%	10.2%	3.6%	16.8%	3.0%
	**True Genes, *pe*FDR = 5%**	99	118	212	241	571	642

**High : mean **τg2 MathType@MTEF@5@5@+=feaafiart1ev1aaatCvAUfKttLearuWrP9MDH5MBPbIqV92AaeXatLxBI9gBaebbnrfifHhDYfgasaacH8akY=wiFfYdH8Gipec8Eeeu0xXdbba9frFj0=OqFfea0dXdd9vqai=hGuQ8kuc9pgc9s8qqaq=dirpe0xb9q8qiLsFr0=vr0=vr0dc8meaabaqaciaacaGaaeqabaqabeGadaaakeaaiiGacqWFepaDdaqhaaWcbaGaem4zaCgabaGaeGOmaidaaaaa@30EE@**= 0.7 (DE); 0.07 (non DE)**	***t*IDR, *γ *= 0.95**	3.8%	13.3%	0%	5.4%	0%	3.4%
	***t*IRR, *γ *= 0.95**	9.8%	0.9%	7.9%	2.8%	10.7%	3.5%
	**True Genes, *pe*FDR = 5%**	122	135	259	270	654	688

**Figure 5:**
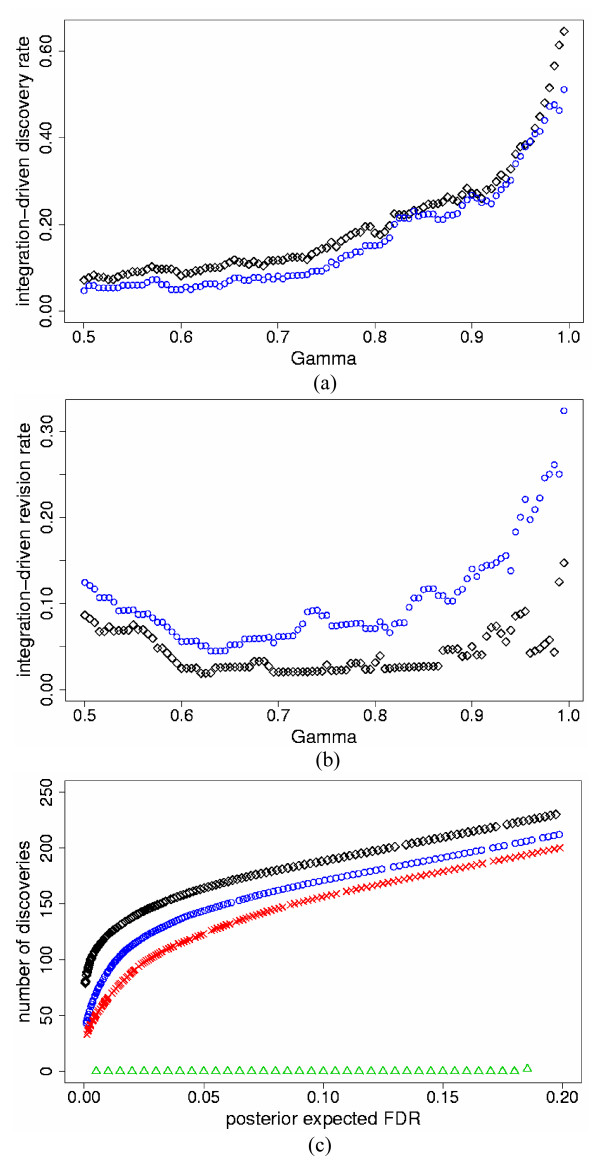
**IDR, IRR and discovered genes versus *pe*FDR for the biological data**. a) Integration-driven discovery rate (IDR) versus threshold values of posterior probability of differential expression *γ *≥ 0.50, for the standardized expression integration model (blue circles) and probability integration model (black diamonds) for the *B. subtilis *mutant and induction biological study data; b) Integration-driven revision rate (IRR) versus threshold values of posterior probability of differential expression *γ *≥ 0.50, for the standardized expression integration model (blue circles) and probability integration model (black diamonds) for the *B. subtilis *mutant and induction biological study data; c) The maximum number of differentially expressed genes versus posterior expected false discovery rate *(peFDR) *for the standardized expression integration model (blue circles), probability integration model (black diamonds), individual analyses of *B. subtilis *mutant study (red checks) and induction study (green triangles).

For all simulated data sets, both the SEI and PI models again identified more true discoveries than the individual analyses for the same levels of *pe*FDR < 20%; the PI model also found more true discoveries than the SEI model, similar to the two-study simulations. Figure 4c displays the results for *p*_*s *_= 10% and high mean τg2
 MathType@MTEF@5@5@+=feaafiart1ev1aaatCvAUfKttLearuWrP9MDH5MBPbIqV92AaeXatLxBI9gBaebbnrfifHhDYfgasaacH8akY=wiFfYdH8Gipec8Eeeu0xXdbba9frFj0=OqFfea0dXdd9vqai=hGuQ8kuc9pgc9s8qqaq=dirpe0xb9q8qiLsFr0=vr0=vr0dc8meaabaqaciaacaGaaeqabaqabeGadaaakeaaiiGacqWFepaDdaqhaaWcbaGaem4zaCgabaGaeGOmaidaaaaa@30EE@ ; Table 2 reports the results for all data sets, with representative *pe*FDR = 5%. When compared to two-study simulations, combining more studies resulted in more true discovered genes for the same levels of *pe*FDR, for both the SEI and PI models. This indicates that pooling more data improves the accuracy of *pe*FDR.

The PI model showed improved performance over the SEI model in simulations of five studies for similar reasons as discussed in the simulations of two studies. By calculating the probability of differential expression based on the separate study means in the PI model, genes with high probability of differential expression in at least one study produce a higher overall probability of differential expression in the PI meta-analysis. However, the SEI model first produces an overall mean and then calculates the probability of differential expression based on this overall mean, which results in fewer true genes being declared differentially expressed. Due to these meta-analytic model differences, the PI model results in higher *t*IDR, lower *t*IRR and more true differentially expressed genes identified for the same level of false discovery than the SEI model.

### Biological data results

We implemented the SEI and PI models to combine the *B. subtilis *mutant and induction studies, using 2,509 genes that had at least one expression value in each study. We also analyzed each study individually. Each slide was standardized to have zero mean and unit standard deviation. Since the truly differentially expressed genes are unknown for the biological data, we report results somewhat differently than for the simulated data. For both models, we show IDR and IRR for fixed levels of *γ *≥ 0.50 with corresponding *pe*FDR (Figures 5a, 5b and Table 3). The PI model produced higher IDR than the SEI model for most levels of *γ *≥ 0.50, with corresponding lower *pe*FDR. In a few instances, the IDR was higher for the SEI than the PI model, but the difference was less than 1%, and the corresponding *pe*FDR was lower for the PI model. On average, the PI model produced an IDR 3.5% higher than the SEI model for *γ *≥ 0.50, and 6.5% higher for *γ *≥ 0.95; the corresponding average *pe*FDR was 1% and 0.3% lower for the PI model, respectively. The IRR was lower for the PI model for all values of *γ *≥ 0.50. In addition, for fixed levels of *pe*FDR < 20%, both the PI and SEI models discovered more genes than the individual studies alone, and the PI model discovered more genes than the SEI model in all cases (Figure 5c).

**Table 3:** Results for *Bacillus subtilis *biological data. Integration-driven discovery rate (IDR), integration-driven revision rate (IRR) and posterior expected false discovery rate (*pe*FDR) for various threshold values of posterior probability of differential expression *γ *≥ 0.50, for the standardized expression integration (SEI) and probability integration (PI) models applied to the *B. subtilis *mutant and induction biological study data.

	**Integration-Driven Discovery Rate**		**Integration-Driven Revision Rate**		**Posterior Expected FDR**	
	
***γ***	**SEI**	**PI**	**SEI**	**PI**	**SEI**	**PI**
**0.995**	51.1%	64.6%	32.4%	14.7%	0.0008	0.0004
**0.99**	46.3%	61.4%	28.2%	12.7%	0.002	0.001
**0.95**	35.7%	37.9%	20.3%	8.9%	0.013	0.007
**0.90**	26.7%	27.1%	7.1%	3.1%	0.022	0.014
**0.50**	4.7%	7.1%	12.4%	8.6%	0.098	0.085

### Sensitivity analysis

The prior distributions of the slide effect and experiment effect variance parameters, φjg2
 MathType@MTEF@5@5@+=feaafiart1ev1aaatCvAUfKttLearuWrP9MDH5MBPbIqV92AaeXatLxBI9gBaebbnrfifHhDYfgasaacH8akY=wiFfYdH8Gipec8Eeeu0xXdbba9frFj0=OqFfea0dXdd9vqai=hGuQ8kuc9pgc9s8qqaq=dirpe0xb9q8qiLsFr0=vr0=vr0dc8meaabaqaciaacaGaaeqabaqabeGadaaakeaaiiGacqWFgpGzdaqhaaWcbaGaemOAaOMaem4zaCgabaGaeGOmaidaaaaa@323F@ and σjg2
 MathType@MTEF@5@5@+=feaafiart1ev1aaatCvAUfKttLearuWrP9MDH5MBPbIqV92AaeXatLxBI9gBaebbnrfifHhDYfgasaacH8akY=wiFfYdH8Gipec8Eeeu0xXdbba9frFj0=OqFfea0dXdd9vqai=hGuQ8kuc9pgc9s8qqaq=dirpe0xb9q8qiLsFr0=vr0=vr0dc8meaabaqaciaacaGaaeqabaqabeGadaaakeaaiiGacqWFdpWCdaqhaaWcbaGaemOAaOMaem4zaCgabaGaeGOmaidaaaaa@3249@, respectively, require some information from the data in order for the models to converge. When assigning uninformative distributions to these parameters, i.e. φjg2
 MathType@MTEF@5@5@+=feaafiart1ev1aaatCvAUfKttLearuWrP9MDH5MBPbIqV92AaeXatLxBI9gBaebbnrfifHhDYfgasaacH8akY=wiFfYdH8Gipec8Eeeu0xXdbba9frFj0=OqFfea0dXdd9vqai=hGuQ8kuc9pgc9s8qqaq=dirpe0xb9q8qiLsFr0=vr0=vr0dc8meaabaqaciaacaGaaeqabaqabeGadaaakeaaiiGacqWFgpGzdaqhaaWcbaGaemOAaOMaem4zaCgabaGaeGOmaidaaaaa@323F@ ~ Inverse Gamma(0.001,0.001) and σjg2
 MathType@MTEF@5@5@+=feaafiart1ev1aaatCvAUfKttLearuWrP9MDH5MBPbIqV92AaeXatLxBI9gBaebbnrfifHhDYfgasaacH8akY=wiFfYdH8Gipec8Eeeu0xXdbba9frFj0=OqFfea0dXdd9vqai=hGuQ8kuc9pgc9s8qqaq=dirpe0xb9q8qiLsFr0=vr0=vr0dc8meaabaqaciaacaGaaeqabaqabeGadaaakeaaiiGacqWFdpWCdaqhaaWcbaGaemOAaOMaem4zaCgabaGaeGOmaidaaaaa@3249@ ~ Inverse Gamma(0.001,0.001), the models do not converge. We thus assigned inverse chi-squared distributions to φjg2
 MathType@MTEF@5@5@+=feaafiart1ev1aaatCvAUfKttLearuWrP9MDH5MBPbIqV92AaeXatLxBI9gBaebbnrfifHhDYfgasaacH8akY=wiFfYdH8Gipec8Eeeu0xXdbba9frFj0=OqFfea0dXdd9vqai=hGuQ8kuc9pgc9s8qqaq=dirpe0xb9q8qiLsFr0=vr0=vr0dc8meaabaqaciaacaGaaeqabaqabeGadaaakeaaiiGacqWFgpGzdaqhaaWcbaGaemOAaOMaem4zaCgabaGaeGOmaidaaaaa@323F@ and σjg2
 MathType@MTEF@5@5@+=feaafiart1ev1aaatCvAUfKttLearuWrP9MDH5MBPbIqV92AaeXatLxBI9gBaebbnrfifHhDYfgasaacH8akY=wiFfYdH8Gipec8Eeeu0xXdbba9frFj0=OqFfea0dXdd9vqai=hGuQ8kuc9pgc9s8qqaq=dirpe0xb9q8qiLsFr0=vr0=vr0dc8meaabaqaciaacaGaaeqabaqabeGadaaakeaaiiGacqWFdpWCdaqhaaWcbaGaemOAaOMaem4zaCgabaGaeGOmaidaaaaa@3249@, with scale parameters based on pooling variance information from all genes, similar to other authors ([17,23,24]). We used 3 degrees of freedom so that the prior distributions were as uninformative as possible (similar to [17]). To examine the sensitivity of the results to the degrees of freedom of the scaled inverse chi-squared distributions, we performed sensitivity analyses for the two-study and five-study simulation data sets as well as the biological data. For the simulation data, we used the data sets with percent of differentially expressed genes *p*_*s *_= 10% and medium mean τg2
 MathType@MTEF@5@5@+=feaafiart1ev1aaatCvAUfKttLearuWrP9MDH5MBPbIqV92AaeXatLxBI9gBaebbnrfifHhDYfgasaacH8akY=wiFfYdH8Gipec8Eeeu0xXdbba9frFj0=OqFfea0dXdd9vqai=hGuQ8kuc9pgc9s8qqaq=dirpe0xb9q8qiLsFr0=vr0=vr0dc8meaabaqaciaacaGaaeqabaqabeGadaaakeaaiiGacqWFepaDdaqhaaWcbaGaem4zaCgabaGaeGOmaidaaaaa@30EE@. We repeated the analyses with the following degrees of freedom for both φjg2
 MathType@MTEF@5@5@+=feaafiart1ev1aaatCvAUfKttLearuWrP9MDH5MBPbIqV92AaeXatLxBI9gBaebbnrfifHhDYfgasaacH8akY=wiFfYdH8Gipec8Eeeu0xXdbba9frFj0=OqFfea0dXdd9vqai=hGuQ8kuc9pgc9s8qqaq=dirpe0xb9q8qiLsFr0=vr0=vr0dc8meaabaqaciaacaGaaeqabaqabeGadaaakeaaiiGacqWFgpGzdaqhaaWcbaGaemOAaOMaem4zaCgabaGaeGOmaidaaaaa@323F@ and σjg2
 MathType@MTEF@5@5@+=feaafiart1ev1aaatCvAUfKttLearuWrP9MDH5MBPbIqV92AaeXatLxBI9gBaebbnrfifHhDYfgasaacH8akY=wiFfYdH8Gipec8Eeeu0xXdbba9frFj0=OqFfea0dXdd9vqai=hGuQ8kuc9pgc9s8qqaq=dirpe0xb9q8qiLsFr0=vr0=vr0dc8meaabaqaciaacaGaaeqabaqabeGadaaakeaaiiGacqWFdpWCdaqhaaWcbaGaemOAaOMaem4zaCgabaGaeGOmaidaaaaa@3249@ : 6, 10, 20, 40. Larger degrees of freedom correspond to more informative priors, i.e. smaller means and variances imposed upon the variance parameters. In all analyses, we found that with more informative priors, the mean posterior probabilities of differential expression for all genes increased. For the two-study simulation data, this resulted in larger numbers of true genes identified for the same levels of *pe*FDR for both the SEI and PI models. *t*IDR decreased for larger degrees of freedom for thresholds of *γ *≥ 0.50 for both the SEI and PI models, since the individual studies as well as the meta-analyses produced higher posterior probabilities of differential expression, which lowered *t*IDR. *t*IRR decreased for larger degrees of freedom for thresholds of *γ *≥ 0.50 for both the SEI and PI models, again due to the higher posterior probabilities of differential expression.

The five-study simulation results were similar to the two-study results, except that the *t*IRR increased slightly for the SEI model for larger degrees of freedom for some thresholds of *γ *≥ 0.50. This was due to the larger number of individual studies; with more individual studies, there were more genes with higher posterior probabilities of differential expression in at least one individual study versus the SEI meta-analysis, which increased *t*IRR for the SEI model. For the biological data, we found similar results to the two-study simulation data: with larger degrees of freedom, IDR, IRR and *pe*FDR decreased on average for thresholds of *γ *≥ 0.50 for both the SEI and PI models.

Overall, the PI model outperformed the SEI model for all prior degrees of freedom imposed, and using 3 degrees of freedom resulted in the most conservative findings for the posterior probabilities of differential expression. We show results for the two-study and five-study simulation data in Table 4 and Supplemental Figures S1 and S2 (see Additional file 1); the biological data results are displayed in Table 5 and Supplemental Figure S3 (see Additional file 1).

**Table 4:** Sensitivity analysis results for the two-study and five-study simulation data. Sensitivity analysis results for the prior degrees of freedom assigned to the slide and experiment effect variance parameters, φjg2
 MathType@MTEF@5@5@+=feaafiart1ev1aaatCvAUfKttLearuWrP9MDH5MBPbIqV92AaeXatLxBI9gBaebbnrfifHhDYfgasaacH8akY=wiFfYdH8Gipec8Eeeu0xXdbba9frFj0=OqFfea0dXdd9vqai=hGuQ8kuc9pgc9s8qqaq=dirpe0xb9q8qiLsFr0=vr0=vr0dc8meaabaqaciaacaGaaeqabaqabeGadaaakeaaiiGacqWFgpGzdaqhaaWcbaGaemOAaOMaem4zaCgabaGaeGOmaidaaaaa@323F@ and σjg2
 MathType@MTEF@5@5@+=feaafiart1ev1aaatCvAUfKttLearuWrP9MDH5MBPbIqV92AaeXatLxBI9gBaebbnrfifHhDYfgasaacH8akY=wiFfYdH8Gipec8Eeeu0xXdbba9frFj0=OqFfea0dXdd9vqai=hGuQ8kuc9pgc9s8qqaq=dirpe0xb9q8qiLsFr0=vr0=vr0dc8meaabaqaciaacaGaaeqabaqabeGadaaakeaaiiGacqWFdpWCdaqhaaWcbaGaemOAaOMaem4zaCgabaGaeGOmaidaaaaa@3249@, respectively, for the two-study and five-study simulation data sets with medium mean τg2
 MathType@MTEF@5@5@+=feaafiart1ev1aaatCvAUfKttLearuWrP9MDH5MBPbIqV92AaeXatLxBI9gBaebbnrfifHhDYfgasaacH8akY=wiFfYdH8Gipec8Eeeu0xXdbba9frFj0=OqFfea0dXdd9vqai=hGuQ8kuc9pgc9s8qqaq=dirpe0xb9q8qiLsFr0=vr0=vr0dc8meaabaqaciaacaGaaeqabaqabeGadaaakeaaiiGacqWFepaDdaqhaaWcbaGaem4zaCgabaGaeGOmaidaaaaa@30EE@ = 0.3 (differentially expressed); 0.03 (non-differentially expressed) and simulated percent differentially expressed genes *p*_*s *_= 10%. Shown are true integration-driven discovery rate (*t*IDR) and true integration-driven revision rate (*t*IRR) for threshold value of posterior probability of differential expression *γ *= 0.95, and the number of true discovered genes for posterior expected false discovery rate *pe*FDR = 5%, for the Bayesian standardized expression integration (SEI) and probability integration (PI) models.

**Two-Study Simulation Data**		**True Integration-Driven Discovery Rate, *γ *= 0.95**		**True Integration-Driven Revision Rate, *γ *= 0.95**		**True Genes, *pe*FDR = 5%**	
**Prior Degrees of Freedom**							
φjg2 MathType@MTEF@5@5@+=feaafiart1ev1aaatCvAUfKttLearuWrP9MDH5MBPbIqV92AaeXatLxBI9gBaebbnrfifHhDYfgasaacH8akY=wiFfYdH8Gipec8Eeeu0xXdbba9frFj0=OqFfea0dXdd9vqai=hGuQ8kuc9pgc9s8qqaq=dirpe0xb9q8qiLsFr0=vr0=vr0dc8meaabaqaciaacaGaaeqabaqabeGadaaakeaaiiGacqWFgpGzdaqhaaWcbaGaemOAaOMaem4zaCgabaGaeGOmaidaaaaa@323F@	σjg2 MathType@MTEF@5@5@+=feaafiart1ev1aaatCvAUfKttLearuWrP9MDH5MBPbIqV92AaeXatLxBI9gBaebbnrfifHhDYfgasaacH8akY=wiFfYdH8Gipec8Eeeu0xXdbba9frFj0=OqFfea0dXdd9vqai=hGuQ8kuc9pgc9s8qqaq=dirpe0xb9q8qiLsFr0=vr0=vr0dc8meaabaqaciaacaGaaeqabaqabeGadaaakeaaiiGacqWFdpWCdaqhaaWcbaGaemOAaOMaem4zaCgabaGaeGOmaidaaaaa@3249@	**SEI**	**PI**	**SEI**	**PI**	**SEI**	**PI**

**3**	**3**	10.8%	16.2%	15.9%	4.5%	185	211
**6**	**6**	4.4%	11.9%	11.6%	1.2%	197	216
**10**	**10**	3.6%	10.2%	7.9%	0.6%	202	218
**20**	**20**	1.7%	8.1%	4.4%	0.5%	206	219
**40**	**40**	1.6%	6.6%	5.9%	0.5%	208	221

**Five-Study Simulation Data**							

**3**	**3**	2.2%	15.2%	10.2%	3.6%	212	241
**6**	**6**	0%	8.8%	13.0%	2.8%	219	246
**10**	**10**	0%	6.9%	13.1%	2.7%	225	248
**20**	**20**	0%	3.9%	14.4%	3.1%	228	250
**40**	**40**	0%	5.1%	12.8%	1.8%	227	250

**Table 5:** Sensitivity analysis results for the *Bacillus subtilis *biological data. Sensitivity analysis results for the prior degrees of freedom assigned to the slide and experiment effect variance parameters, φjg2
 MathType@MTEF@5@5@+=feaafiart1ev1aaatCvAUfKttLearuWrP9MDH5MBPbIqV92AaeXatLxBI9gBaebbnrfifHhDYfgasaacH8akY=wiFfYdH8Gipec8Eeeu0xXdbba9frFj0=OqFfea0dXdd9vqai=hGuQ8kuc9pgc9s8qqaq=dirpe0xb9q8qiLsFr0=vr0=vr0dc8meaabaqaciaacaGaaeqabaqabeGadaaakeaaiiGacqWFgpGzdaqhaaWcbaGaemOAaOMaem4zaCgabaGaeGOmaidaaaaa@323F@ and σjg2
 MathType@MTEF@5@5@+=feaafiart1ev1aaatCvAUfKttLearuWrP9MDH5MBPbIqV92AaeXatLxBI9gBaebbnrfifHhDYfgasaacH8akY=wiFfYdH8Gipec8Eeeu0xXdbba9frFj0=OqFfea0dXdd9vqai=hGuQ8kuc9pgc9s8qqaq=dirpe0xb9q8qiLsFr0=vr0=vr0dc8meaabaqaciaacaGaaeqabaqabeGadaaakeaaiiGacqWFdpWCdaqhaaWcbaGaemOAaOMaem4zaCgabaGaeGOmaidaaaaa@3249@, respectively, for the *B. subtilis *mutant and induction biological study data. Shown are the integration-driven discovery rate (IDR), integration-driven revision rate (IRR) and posterior expected false discovery rate (*pe*FDR) for threshold value of posterior probability of differential expression *γ *= 0.95, for the standardized expression integration (SEI) and probability integration (PI) models applied to the *B. subtilis *mutant and induction biological study data.

**Prior Degrees of Freedom**		**Integration-Driven Discovery Rate, *γ *= 0.95**		**Integration-Driven Revision Rate, *γ *= 0.95**		**Posterior Expected FDR, *γ *= 0.95**	
		
φjg2 MathType@MTEF@5@5@+=feaafiart1ev1aaatCvAUfKttLearuWrP9MDH5MBPbIqV92AaeXatLxBI9gBaebbnrfifHhDYfgasaacH8akY=wiFfYdH8Gipec8Eeeu0xXdbba9frFj0=OqFfea0dXdd9vqai=hGuQ8kuc9pgc9s8qqaq=dirpe0xb9q8qiLsFr0=vr0=vr0dc8meaabaqaciaacaGaaeqabaqabeGadaaakeaaiiGacqWFgpGzdaqhaaWcbaGaemOAaOMaem4zaCgabaGaeGOmaidaaaaa@323F@	σjg2 MathType@MTEF@5@5@+=feaafiart1ev1aaatCvAUfKttLearuWrP9MDH5MBPbIqV92AaeXatLxBI9gBaebbnrfifHhDYfgasaacH8akY=wiFfYdH8Gipec8Eeeu0xXdbba9frFj0=OqFfea0dXdd9vqai=hGuQ8kuc9pgc9s8qqaq=dirpe0xb9q8qiLsFr0=vr0=vr0dc8meaabaqaciaacaGaaeqabaqabeGadaaakeaaiiGacqWFdpWCdaqhaaWcbaGaemOAaOMaem4zaCgabaGaeGOmaidaaaaa@3249@	**SEI**	**PI**	**SEI**	**PI**	**SEI**	**PI**
**3**	**3**	35.7%	37.9%	20.3%	8.9%	0.013	0.0073
**6**	**6**	13.9%	20.1%	7.9%	5.3%	0.0088	0.0049
**10**	**10**	7.1%	14.0%	7.1%	2.4%	0.0059	0.0037
**20**	**20**	6.3%	14.5%	7.7%	0.0%	0.0044	0.0040
**40**	**40**	5.3%	13.3%	10.1%	0.7%	0.0038	0.0041

## Conclusion

We compared two Bayesian approaches to meta-analysis of microarray data: the standardized expression integration and probability integration models. The standardized expression integration model includes inter-study variability and may thus improve accuracy of findings; it also produces an overall estimate of standardized gene expression among all studies. However, due to the typical small number of studies in meta-analyses for microarrays, the inter-study variability is difficult to model. Alternatively, the probability integration approach eliminates the need to specify inter-study variability since each study is modeled separately, with overall smoothing of probabilities across studies. In our simulations of two and five studies, the probability integration model produced higher *t*IDR and lower *t*IRR than the standardized expression integration model for fixed posterior probabilities of differential expression, and also identified more true discoveries for the same levels of *pe*FDR. We found similar results for the biological data, with the probability integration model producing higher IDR on average and lower IRR with corresponding lower values of *pe*FDR, for fixed probabilities of differential expression. We conclude that, for our data sets, aggregating probabilities across studies rather than combining gene expression levels improves IDR, IRR and the number of discovered genes versus *peFDR*.

In the standardized expression integration meta-analysis model, the prior assignment for the inter-study variability had a large impact on the results. We assigned some of the most common prior distributions used in practice: inverse gamma, log-logistic and locally uniform. The uninformative inverse gamma and log-logistic distributions were gene-specific and did not pool information from similar genes; these distributions did not improve results versus individual analyses. The informative inverse gamma distributions pooled information either from all genes or sets of differentially and non-differentially expression genes, but also did not improve upon separate study analyses. We found the most improvement in true integration-driven discovery rates and increases in true discoveries versus *pe*FDR using the locally uniform prior distribution centered at the medians of the differentially expressed and non-expressed genes; this emphasizes the need for priors that pool information across genes rather than using individual gene information or uninformative priors, for small data sets.

The probability integration model does not produce an overall measure of expression for each gene, similar to the classical meta-analysis methods of combining *p*-values, probabilities and ranks. However, study-specific gene expression values are produced, and these can be examined for individual genes of interest.

The standardized expression integration and probability integration models presented here were developed for studies from the same platform. A common control sample is not required across studies, and the studies are assumed to be independent. In addition, the models do not require the same array-layout across studies. For example, some studies could have replicate slides within repeated experiments, while other studies could have only replicate slides within a single study. The models are thus applicable to a wide range of study designs.

## Methods

### Two study simulation data

We simulated data for two studies, with similar format to the *B. subtilis *mutant and induction biological data (see Methods: Biological data), with simulated proportion of differentially expressed genes *p*_*s *_= 5%, 10%, 25%. Each study contained 3,000 genes. Study 1 had 5 replicate slides within 3 repeated experiments, and Study 2 had 4 replicate slides within 3 repeated experiments. We simulated data from Model (1), with model parameters chosen to resemble the biological data. We set ηg02
 MathType@MTEF@5@5@+=feaafiart1ev1aaatCvAUfKttLearuWrP9MDH5MBPbIqV92AaeXatLxBI9gBaebbnrfifHhDYfgasaacH8akY=wiFfYdH8Gipec8Eeeu0xXdbba9frFj0=OqFfea0dXdd9vqai=hGuQ8kuc9pgc9s8qqaq=dirpe0xb9q8qiLsFr0=vr0=vr0dc8meaabaqaciaacaGaaeqabaqabeGadaaakeaaiiGacqWF3oaAdaqhaaWcbaGaem4zaCMaeGimaadabaGaeGOmaidaaaaa@31C3@ = 0.025 and *c *= 48. For Study 1, we assigned variance across slides to 0.074 and across experiments to 0.026. For Study 2, we assigned slide variation to 0.023 and experiment variation to 0.022. The biological data had inter-study variability that was approximately Normally distributed with mean of 0.33 and variation of 0.12 for the top 10% of genes, and mean 0.031 and variation 0.004 for the remaining genes. We simulated τg2
 MathType@MTEF@5@5@+=feaafiart1ev1aaatCvAUfKttLearuWrP9MDH5MBPbIqV92AaeXatLxBI9gBaebbnrfifHhDYfgasaacH8akY=wiFfYdH8Gipec8Eeeu0xXdbba9frFj0=OqFfea0dXdd9vqai=hGuQ8kuc9pgc9s8qqaq=dirpe0xb9q8qiLsFr0=vr0=vr0dc8meaabaqaciaacaGaaeqabaqabeGadaaakeaaiiGacqWFepaDdaqhaaWcbaGaem4zaCgabaGaeGOmaidaaaaa@30EE@ from a Normal distribution, with mean values that were lower, similar to, and higher than these values for the overexpressed and non-expressed genes, respectively: low mean: 0.1 and 0.01; medium mean: 0.3 and 0.03; high mean: 0.7 and 0.07; with variation values equivalent to the biological data. We refer to these as low, medium and high mean levels of inter-study variability. Each slide was standardized to have mean zero and unit standard deviation. Although correlation of expression is expected among genes, this has been shown to be difficult to simulate. We thus assumed independence among genes in simulations, similar to other studies (see, for example, Gottardo *et al*. [23], Lönnstedt and Speed [24]).

### Simulation data for five studies

For the simulation of five studies, Study 1 and Study 2 were the same as in the previous section, and we simulated data for 3 additional studies, with proportion of differentially expressed genes *p*_*s *_= 5%, 10%, 25%. In total there were 3,000 genes. For Studies 3, 4 and 5, we used the study format similar to Study 1, with 5 replicate slides within 3 replicate experiments. For the slide and experiment variance parameters, we assigned values that were either within the range of values for Study 1 and Study 2, or somewhat outside the range. For Study 3, the slide variance was assigned 0.05, and experiment variance 0.02. For Study 4, the slide variance was assigned 0.04, and experiment variance 0.022. For Study 5, the slide variance was set to 0.06, and experiment variance 0.03. We again set ηg02
 MathType@MTEF@5@5@+=feaafiart1ev1aaatCvAUfKttLearuWrP9MDH5MBPbIqV92AaeXatLxBI9gBaebbnrfifHhDYfgasaacH8akY=wiFfYdH8Gipec8Eeeu0xXdbba9frFj0=OqFfea0dXdd9vqai=hGuQ8kuc9pgc9s8qqaq=dirpe0xb9q8qiLsFr0=vr0=vr0dc8meaabaqaciaacaGaaeqabaqabeGadaaakeaaiiGacqWF3oaAdaqhaaWcbaGaem4zaCMaeGimaadabaGaeGOmaidaaaaa@31C3@ = 0.025 and *c *= 48 and simulated τg2
 MathType@MTEF@5@5@+=feaafiart1ev1aaatCvAUfKttLearuWrP9MDH5MBPbIqV92AaeXatLxBI9gBaebbnrfifHhDYfgasaacH8akY=wiFfYdH8Gipec8Eeeu0xXdbba9frFj0=OqFfea0dXdd9vqai=hGuQ8kuc9pgc9s8qqaq=dirpe0xb9q8qiLsFr0=vr0=vr0dc8meaabaqaciaacaGaaeqabaqabeGadaaakeaaiiGacqWFepaDdaqhaaWcbaGaem4zaCgabaGaeGOmaidaaaaa@30EE@ from Normal distributions with three mean levels: low, medium and high, with variation values equivalent to the biological data, similar to the two-study simulation data. Each slide was standardized to have zero mean and unit standard deviation.

### Biological data

*B. subtilis *is a bacterium that responds to starvation by forming spores, which allow it to survive in extreme environmental conditions. Two independent *B. subtilis *microarray studies were designed to identify genes in the sporulation pathway controlled by the sigma factor *σ*^*E*^. The studies had reciprocal designs; the first was a mutation of *σ*^*E*^, and the second was an induction of *σ*^*E*^, with details in the following (see also [50,58]).

#### Mutant study

For the mutant study, cells with a deletion in the gene for *σ*^*E *^(the mutant sample) were compared to wild-type cells. The wild-type/mutant ratios identified genes under the control of *σ*^*E*^. In total, five microarrays were created from three independent repeated experiments; the first experiment produced three replicate slides and the second and third experiments each produced one slide. Each array contained somewhat more spots than the *B. subtilis *genome size of 4,106 due to multiple spotting of specific genes on the arrays. The percent of missing data due to low quality spots ranged from 18.6% to 64.5% across the five slides; a total of 3,713 genes had measurable expression values for at least one slide. We used log-ratios of intensities [44], normalized slides using a rank-invariant method [17,59] and standardized each slide to have zero mean and unit standard deviation.

#### Induction study

The induction study was an overexpression of *σ*^*E *^in which cells treated with an inducer were compared to unaltered cells. The induction/wild-type ratios identified genes in the *σ*^*E *^regulon. In total, four microarrays were created from three independent repeated experiments. The first two experiments each produced one slide and the third experiment produced two replicate slides. The percent of data removed due to low quality spots ranged from 52.6% to 67.0% across the four slides; a total of 2,552 had measurable expression for at least one slide. We again analyzed the post-normalized log-ratios of intensities, and standardized each array to have zero mean and unit standard deviation.

### Standardized expression integration model: prior distributions for inter-study variation

We assigned the following inverse gamma, log-logistic and locally uniform prior distributions for the inter-study variability parameter τg2
 MathType@MTEF@5@5@+=feaafiart1ev1aaatCvAUfKttLearuWrP9MDH5MBPbIqV92AaeXatLxBI9gBaebbnrfifHhDYfgasaacH8akY=wiFfYdH8Gipec8Eeeu0xXdbba9frFj0=OqFfea0dXdd9vqai=hGuQ8kuc9pgc9s8qqaq=dirpe0xb9q8qiLsFr0=vr0=vr0dc8meaabaqaciaacaGaaeqabaqabeGadaaakeaaiiGacqWFepaDdaqhaaWcbaGaem4zaCgabaGaeGOmaidaaaaa@30EE@ in the standardized expression integration model (Model (1)) for both the simulation and biological data. We applied both uninformative and informative priors; the informative priors either used individual gene information or pooled information from sets of genes. We found that uninformative priors and priors that used individual gene information did not improve upon individual study analyses. Only one prior distribution produced true integration-driven discovery rates > 0% for *γ *≥ 0.50, and an increase in true discoveries versus *pe*FDR < 20% compared to individual study analyses in the two and five study simulations: **3**) locally uniform centered at the median of the inter-study variability (see below).

**1**) Inverse gamma distribution (Choi *et al*. [2], Smith *et al*. [34], Normand [35], Sargent *et al*. [38]): this is a standard conjugate prior distribution for variance parameters. We first applied an uninformative prior distribution, in order to allow the data to inform on the posterior distribution. However, since this distribution did not result in improved performance, we also applied two informative distributions, as follows.

**a) **Uninformative inverse gamma distribution: *IG*(0.001,0.001), corresponding to mean 1, variance 1000.

**b) **Informative inverse gamma distribution: *IG*(*α, β*), with *α*, *β *chosen so that the mean and variance were equal to the mean and variance of the inter-study variability based on data of all genes. This prior distribution pools data from all genes in order to provide a more accurate measure of inter-study variability. Pooling variance information from all genes is similar to the methods of Tseng *et al*. [17], Gottardo *et al*. [23] and Lönnstedt and Speed [24].

**c) **Informative inverse gamma distribution: separate priors for differentially and non-differentially expressed genes. Since the inter-study variability is higher for differentially expressed genes than non-expressed genes, we assigned two different priors conditioned on *I*_*g *_= 1 and *I*_*g *_= 0. For *I*_*g *_= 1, we assigned an inverse-gamma distribution with mean and variance equal to that of the inter-study variability based on the top *p*% of data. The proportion *p *was estimated from the average of the individual study analyses. Similarly for *I*_*g *_= 0, we assigned the prior based on the remaining (1-*p*)% of data. Estimating inverse-gamma parameters separately for the top and remaining proportions of genes is similar to the method of Gottardo *et al*. [23].

**2) **Log-logistic distribution (DuMouchel and Normand [36]):

p(τg)=sg0(sg0+τg)2 for τg≥0, where sg02=K∑sgj−2.
 MathType@MTEF@5@5@+=feaafiart1ev1aaatCvAUfKttLearuWrP9MDH5MBPbIqV92AaeXatLxBI9gBaebbnrfifHhDYfgasaacH8akY=wiFfYdH8Gipec8Eeeu0xXdbba9frFj0=OqFfea0dXdd9vqai=hGuQ8kuc9pgc9s8qqaq=dirpe0xb9q8qiLsFr0=vr0=vr0dc8meaabaqaciaacaGaaeqabaqabeGadaaakeaacqWGWbaCcqGGOaakiiGacqWFepaDdaWgaaWcbaGaem4zaCgabeaakiabcMcaPiabg2da9maalaaabaGaem4Cam3aaSbaaSqaaiabdEgaNjabicdaWaqabaaakeaacqGGOaakcqWGZbWCdaWgaaWcbaGaem4zaCMaeGimaadabeaakiabgUcaRiab=r8a0naaBaaaleaacqWGNbWzaeqaaOGaeiykaKYaaWbaaSqabeaacqaIYaGmaaaaaOGaeeiiaaIaeeOzayMaee4Ba8MaeeOCaiNaeeiiaaIae8hXdq3aaSbaaSqaaiabdEgaNbqabaGccqGHLjYScqaIWaamcqGGSaalcqqGGaaicqqG3bWDcqqGObaAcqqGLbqzcqqGYbGCcqqGLbqzcqqGGaaicqWGZbWCdaqhaaWcbaGaem4zaCMaeGimaadabaGaeGOmaidaaOGaeyypa0ZaaSaaaeaacqWGlbWsaeaadaaeabqaaiabdohaZnaaDaaaleaacqWGNbWzcqWGQbGAaeaacqGHsislcqaIYaGmaaaabeqab0GaeyyeIuoaaaGccqGGUaGlaaa@6845@

Here, *K *is the number of studies and sgj2
 MathType@MTEF@5@5@+=feaafiart1ev1aaatCvAUfKttLearuWrP9MDH5MBPbIqV92AaeXatLxBI9gBaebbnrfifHhDYfgasaacH8akY=wiFfYdH8Gipec8Eeeu0xXdbba9frFj0=OqFfea0dXdd9vqai=hGuQ8kuc9pgc9s8qqaq=dirpe0xb9q8qiLsFr0=vr0=vr0dc8meaabaqaciaacaGaaeqabaqabeGadaaakeaacqWGZbWCdaqhaaWcbaGaem4zaCMaemOAaOgabaGaeGOmaidaaaaa@31EE@ is sampling variability for gene *g *in study *j*.  Note that this prior assignment is calculated for each gene individually. Our data included slide and experiment variance; we thus calculated sgj2
 MathType@MTEF@5@5@+=feaafiart1ev1aaatCvAUfKttLearuWrP9MDH5MBPbIqV92AaeXatLxBI9gBaebbnrfifHhDYfgasaacH8akY=wiFfYdH8Gipec8Eeeu0xXdbba9frFj0=OqFfea0dXdd9vqai=hGuQ8kuc9pgc9s8qqaq=dirpe0xb9q8qiLsFr0=vr0=vr0dc8meaabaqaciaacaGaaeqabaqabeGadaaakeaacqWGZbWCdaqhaaWcbaGaem4zaCMaemOAaOgabaGaeGOmaidaaaaa@31EE@ for gene *g *and study *j *as follows:

sgj2=n1ν12+n2ν22+...+nEνE2(n1+n2+...+nE)2,
 MathType@MTEF@5@5@+=feaafiart1ev1aaatCvAUfKttLearuWrP9MDH5MBPbIqV92AaeXatLxBI9gBaebbnrfifHhDYfgasaacH8akY=wiFfYdH8Gipec8Eeeu0xXdbba9frFj0=OqFfea0dXdd9vqai=hGuQ8kuc9pgc9s8qqaq=dirpe0xb9q8qiLsFr0=vr0=vr0dc8meaabaqaciaacaGaaeqabaqabeGadaaakeaacqWGZbWCdaqhaaWcbaGaem4zaCMaemOAaOgabaGaeGOmaidaaOGaeyypa0ZaaSaaaeaacqWGUbGBdaWgaaWcbaGaeGymaedabeaaiiGakiab=17aUnaaDaaaleaacqaIXaqmaeaacqaIYaGmaaGccqGHRaWkcqWGUbGBdaWgaaWcbaGaeGOmaidabeaakiab=17aUnaaDaaaleaacqaIYaGmaeaacqaIYaGmaaGccqGHRaWkcqGGUaGlcqGGUaGlcqGGUaGlcqGHRaWkcqWGUbGBdaWgaaWcbaGaemyraueabeaakiab=17aUnaaDaaaleaacqWGfbqraeaacqaIYaGmaaaakeaacqGGOaakcqWGUbGBdaWgaaWcbaGaeGymaedabeaakiabgUcaRiabd6gaUnaaBaaaleaacqaIYaGmaeqaaOGaey4kaSIaeiOla4IaeiOla4IaeiOla4Iaey4kaSIaemOBa42aaSbaaSqaaiabdweafbqabaGccqGGPaqkdaahaaWcbeqaaiabikdaYaaaaaGccqGGSaalaaa@5C8E@

where *E *is the total number of experiments, *n*_*e *_is the number of slides within experiment *e*, and νe2
 MathType@MTEF@5@5@+=feaafiart1ev1aaatCvAUfKttLearuWrP9MDH5MBPbIqV92AaeXatLxBI9gBaebbnrfifHhDYfgasaacH8akY=wiFfYdH8Gipec8Eeeu0xXdbba9frFj0=OqFfea0dXdd9vqai=hGuQ8kuc9pgc9s8qqaq=dirpe0xb9q8qiLsFr0=vr0=vr0dc8meaabaqaciaacaGaaeqabaqabeGadaaakeaaiiGacqWF9oGBdaqhaaWcbaGaemyzaugabaGaeGOmaidaaaaa@30DD@ is the sample variance of the *y*_*jgse *_within experiment *e*.

As discussed in DuMouchel and Normand [36], the sg02
 MathType@MTEF@5@5@+=feaafiart1ev1aaatCvAUfKttLearuWrP9MDH5MBPbIqV92AaeXatLxBI9gBaebbnrfifHhDYfgasaacH8akY=wiFfYdH8Gipec8Eeeu0xXdbba9frFj0=OqFfea0dXdd9vqai=hGuQ8kuc9pgc9s8qqaq=dirpe0xb9q8qiLsFr0=vr0=vr0dc8meaabaqaciaacaGaaeqabaqabeGadaaakeaacqWGZbWCdaqhaaWcbaGaem4zaCMaeGimaadabaGaeGOmaidaaaaa@317F@ is the harmonic mean of the *K *sampling variances, sgj2
 MathType@MTEF@5@5@+=feaafiart1ev1aaatCvAUfKttLearuWrP9MDH5MBPbIqV92AaeXatLxBI9gBaebbnrfifHhDYfgasaacH8akY=wiFfYdH8Gipec8Eeeu0xXdbba9frFj0=OqFfea0dXdd9vqai=hGuQ8kuc9pgc9s8qqaq=dirpe0xb9q8qiLsFr0=vr0=vr0dc8meaabaqaciaacaGaaeqabaqabeGadaaakeaacqWGZbWCdaqhaaWcbaGaem4zaCMaemOAaOgabaGaeGOmaidaaaaa@31EE@, and the density *p*(*τ*_*g*_) has median equal to *s*_*g0*_. The quartiles of the distribution of *τ*_*g *_are *s*_*g0*_/3, *s*_*g0 *_and 3*s*_*g0*_, so that the distribution covers a sensible range of values. If the sample standard deviations are not equal across microarray studies, then *s*_*g0 *_will be weighted toward the studies with smaller *s*_*gj *_; this prevents the meta-analysis results from being overly influenced by a few studies that have large *s*_*gj *_values.

**3) **Locally uniform distribution (Gelman *et al*. [39]): separate priors for differentially and non-differentially expressed genes. The uniform prior is used when a variable is known to lie within a specific interval and is equally likely to be found anywhere within the interval. For inter-study variability of gene expression, this prior assignment pools information from the differentially and non-differentially expressed genes in order to provide a more accurate estimate of the variability. Again, since the inter-study variability is higher for differentially expressed than non-expressed genes, we assigned different priors conditioned on *I*_*g *_= 1 and *I*_*g *_= 0. For *I*_*g *_= 1, we assigned a locally uniform prior centered at the median inter-study variability based on the top *p*% of data. The percent *p *was determined from the average of the individual study analyses. Similarly for *I*_*g *_= 0, we assigned a locally uniform prior based on the remaining (1-*p*)% of data. The ranges of the uniform distributions were ± one standard error of the median. The standard error of the median was estimated as 1.253 × standard error of the mean (Kendall *et al*. [60]). We selected the range of ± one standard error of the median based on exploratory calculations. In simulations, this range produced higher *t*IDR for *γ *≥ 0.50 and more true discoveries for levels of *pe*FDR < 20% versus individual study analyses compared to the following alternative ranges: fixed medians (range of zero); and ± two standard errors of the medians. We also repeated prior assignment **3) **using means in place of medians, but this prior specification did not result in improvements in discoveries versus individual study analyses.

#### Normal versus *t*-distribution modeling of the study-specific mean values

As discussed and implemented by several authors (Smith *et al*. [34], Sargent *et al*. [38], Choi *et al*. [2]), an alternative to the Normal distribution assumption for the study-specific mean values *θ*_*jg *_is to model the values as *t*-distributions, due to the small number of studies. We repeated each of the prior assignments **1) **to **3) **above assuming a *t*-distribution with degrees of freedom less than 30 (with degrees of freedom 30 equivalent to a Normal distribution). For simulated data, we found that the *t*IDR versus *γ *≥ 0.50 and the number of true discoveries versus *pe*FDR decreased with fewer degrees of freedom. Based on these findings, we model the mean values *θ*_*jg *_as Normal distributions in the standardized expression integration model. The Normal assumption is commonly used in hierarchical Bayesian meta-analysis models, even for small numbers of studies (see, for example, Normand [35], Pauler and Wakefield [37], Sargent *et al*. [38]).

### Markov chain Monte Carlo implementation

We implement a Markov chain Monte Carlo (MCMC) procedure to simulate the posterior distributions of each of the parameters. MCMC methods generate samples from a density *p*(*ψ*) for a parameter *ψ *(with *p*(*ψ*) possibly known only to a constant of proportionality) by creating a Markov chain on the state space of *ψ *which has *p *as its true (stationary) distribution (see Liu *et al*. [51] for further details).

#### Joint posterior distributions

For Model (1), the joint distribution of the data and parameters is:

p(yjgse,ξjge,φjg2,σjg2,θjg,τg2,μg,Ig,p,ηg02,c)=[∏j=1J∏g=1G∏e=1E{∏s=1Sep(yjgse|ξjge,φjg2)p(ξjge|θjg,σjg2)}p(θjg|μg,τg2)p(μg,Ig|p,Ωj)×p(φjg2)p(σjg2)p(τg2)p(p)p(Ωj)],
 MathType@MTEF@5@5@+=feaafiart1ev1aaatCvAUfKttLearuWrP9MDH5MBPbIqV92AaeXatLxBI9gBaebbnrfifHhDYfgasaacH8akY=wiFfYdH8Gipec8Eeeu0xXdbba9frFj0=OqFfea0dXdd9vqai=hGuQ8kuc9pgc9s8qqaq=dirpe0xb9q8qiLsFr0=vr0=vr0dc8meaabaqaciaacaGaaeqabaqabeGadaaakeaafaqaaeWabaaabaGaemiCaaNaeiikaGIaemyEaK3aaSbaaSqaaiabdQgaQjabdEgaNjabdohaZjabdwgaLbqabaGccqGGSaaliiGacqWF+oaEdaWgaaWcbaGaemOAaOMaem4zaCMaemyzaugabeaakiabcYcaSiab=z8aMnaaDaaaleaacqWGQbGAcqWGNbWzaeaacqaIYaGmaaGccqGGSaalcqWFdpWCdaqhaaWcbaGaemOAaOMaem4zaCgabaGaeGOmaidaaOGaeiilaWIae8hUde3aaSbaaSqaaiabdQgaQjabdEgaNbqabaGccqGGSaalcqWFepaDdaqhaaWcbaGaem4zaCgabaGaeGOmaidaaOGaeiilaWIae8hVd02aaSbaaSqaaiabdEgaNbqabaGccqGGSaalcqWGjbqsdaWgaaWcbaGaem4zaCgabeaakiabcYcaSiabdchaWjabcYcaSiab=D7aOnaaDaaaleaacqWGNbWzcqaIWaamaeaacqaIYaGmaaGccqGGSaalcqWGJbWycqGGPaqkcqGH9aqpaeaadaWabaqaamaarahabaWaaebCaeaadaqeWbqaamaacmqabaWaaebCaeaacqWGWbaCcqGGOaakcqWG5bqEdaWgaaWcbaGaemOAaOMaem4zaCMaem4CamNaemyzaugabeaakiabcYha8jab=57a4naaBaaaleaacqWGQbGAcqWGNbWzcqWGLbqzaeqaaOGaeiilaWIae8NXdy2aa0baaSqaaiabdQgaQjabdEgaNbqaaiabikdaYaaakiabcMcaPiabdchaWjabcIcaOiab=57a4naaBaaaleaacqWGQbGAcqWGNbWzcqWGLbqzaeqaaOGaeiiFaWNae8hUde3aaSbaaSqaaiabdQgaQjabdEgaNbqabaGccqGGSaalcqWFdpWCdaqhaaWcbaGaemOAaOMaem4zaCgabaGaeGOmaidaaOGaeiykaKcaleaacqWGZbWCcqGH9aqpcqaIXaqmaeaacqWGtbWudaWgaaadbaGaemyzaugabeaaa0Gaey4dIunaaOGaay5Eaiaaw2haaiabdchaWjabcIcaOiab=H7aXnaaBaaaleaacqWGQbGAcqWGNbWzaeqaaOGaeiiFaWNae8hVd02aaSbaaSqaaiabdEgaNbqabaGccqGGSaalcqWFepaDdaqhaaWcbaGaem4zaCgabaGaeGOmaidaaOGaeiykaKIaemiCaaNaeiikaGIae8hVd02aaSbaaSqaaiabdEgaNbqabaGccqGGSaalcqWGjbqsdaWgaaWcbaGaem4zaCgabeaakiabcYha8jabdchaWjabcYcaSGGabiab+L6axnaaBaaaleaaieqacqqFQbGAaeqaaOGaeiykaKIaey41aqlaleaacqWGLbqzcqGH9aqpcqaIXaqmaeaacqWGfbqra0Gaey4dIunaaSqaaiabdEgaNjabg2da9iabigdaXaqaaiabdEeahbqdcqGHpis1aaWcbaGaemOAaOMaeyypa0JaeGymaedabaGaemOsaOeaniabg+GivdaakiaawUfaaaqaamaadiaabaGaemiCaaNaeiikaGIae8NXdy2aa0baaSqaaiabdQgaQjabdEgaNbqaaiabikdaYaaakiabcMcaPiabdchaWjabcIcaOiab=n8aZnaaDaaaleaacqWGQbGAcqWGNbWzaeaacqaIYaGmaaGccqGGPaqkcqWGWbaCcqGGOaakcqWFepaDdaqhaaWcbaGaem4zaCgabaGaeGOmaidaaOGaeiykaKIaemiCaaNaeiikaGIaemiCaaNaeiykaKIaemiCaaNaeiikaGIae4xQdC1aaSbaaSqaaiab9PgaQbqabaGccqGGPaqkaiaaw2faaiabcYcaSaaaaaa@FF72@

where **Ω**_**j **_= (ηg02
 MathType@MTEF@5@5@+=feaafiart1ev1aaatCvAUfKttLearuWrP9MDH5MBPbIqV92AaeXatLxBI9gBaebbnrfifHhDYfgasaacH8akY=wiFfYdH8Gipec8Eeeu0xXdbba9frFj0=OqFfea0dXdd9vqai=hGuQ8kuc9pgc9s8qqaq=dirpe0xb9q8qiLsFr0=vr0=vr0dc8meaabaqaciaacaGaaeqabaqabeGadaaakeaaiiGacqWF3oaAdaqhaaWcbaGaem4zaCMaeGimaadabaGaeGOmaidaaaaa@31C3@, *c*), *j *= study, *g *= gene, *e *= experiment, *s *= slide.

For Model (2), the joint distribution of the data and parameters is:

p(yjgse,ξjge,φjg2,σjg2,θjg,Ig,p,ηjg02,cj)=∏j=1J∏g=1G∏e=1E{∏s=1Sep(yjgse|ξjge,φjg2)p(ξjge|θjg,σjg2)}p(θjg,Ig|p,Ωj)p(φjg2)p(σjg2)p(p)p(Ωj),
 MathType@MTEF@5@5@+=feaafiart1ev1aaatCvAUfKttLearuWrP9MDH5MBPbIqV92AaeXatLxBI9gBaebbnrfifHhDYfgasaacH8akY=wiFfYdH8Gipec8Eeeu0xXdbba9frFj0=OqFfea0dXdd9vqai=hGuQ8kuc9pgc9s8qqaq=dirpe0xb9q8qiLsFr0=vr0=vr0dc8meaabaqaciaacaGaaeqabaqabeGadaaakeaafaqaaeGabaaabaGaemiCaaNaeiikaGIaemyEaK3aaSbaaSqaaiabdQgaQjabdEgaNjabdohaZjabdwgaLbqabaGccqGGSaaliiGacqWF+oaEdaWgaaWcbaGaemOAaOMaem4zaCMaemyzaugabeaakiabcYcaSiab=z8aMnaaDaaaleaacqWGQbGAcqWGNbWzaeaacqaIYaGmaaGccqGGSaalcqWFdpWCdaqhaaWcbaGaemOAaOMaem4zaCgabaGaeGOmaidaaOGaeiilaWIae8hUde3aaSbaaSqaaiabdQgaQjabdEgaNbqabaGccqGGSaalcqWGjbqsdaWgaaWcbaGaem4zaCgabeaakiabcYcaSiabdchaWjabcYcaSiab=D7aOnaaDaaaleaacqWGQbGAcqWGNbWzcqaIWaamaeaacqaIYaGmaaGccqGGSaalcqWGJbWydaWgaaWcbaGaemOAaOgabeaakiabcMcaPiabg2da9aqaamaarahabaWaaebCaeaadaqeWbqaamaacmqabaWaaebCaeaacqWGWbaCcqGGOaakcqWG5bqEdaWgaaWcbaGaemOAaOMaem4zaCMaem4CamNaemyzaugabeaakiabcYha8jab=57a4naaBaaaleaacqWGQbGAcqWGNbWzcqWGLbqzaeqaaOGaeiilaWIae8NXdy2aa0baaSqaaiabdQgaQjabdEgaNbqaaiabikdaYaaakiabcMcaPiabdchaWjabcIcaOiab=57a4naaBaaaleaacqWGQbGAcqWGNbWzcqWGLbqzaeqaaOGaeiiFaWNae8hUde3aaSbaaSqaaiabdQgaQjabdEgaNbqabaGccqGGSaalcqWFdpWCdaqhaaWcbaGaemOAaOMaem4zaCgabaGaeGOmaidaaOGaeiykaKcaleaacqWGZbWCcqGH9aqpcqaIXaqmaeaacqWGtbWudaWgaaadbaGaemyzaugabeaaa0Gaey4dIunaaOGaay5Eaiaaw2haaiabdchaWjabcIcaOiab=H7aXnaaBaaaleaacqWGQbGAcqWGNbWzaeqaaOGaeiilaWIaemysaK0aaSbaaSqaaiabdEgaNbqabaGccqGG8baFcqWGWbaCcqGGSaaliiqacqGFPoWvdaWgaaWcbaacbeGae0NAaOgabeaakiabcMcaPiabdchaWjabcIcaOiab=z8aMnaaDaaaleaacqWGQbGAcqWGNbWzaeaacqaIYaGmaaGccqGGPaqkcqWGWbaCcqGGOaakcqWFdpWCdaqhaaWcbaGaemOAaOMaem4zaCgabaGaeGOmaidaaOGaeiykaKIaemiCaaNaeiikaGIaemiCaaNaeiykaKIaemiCaaNaeiikaGIae4xQdC1aaSbaaSqaaiab9PgaQbqabaGccqGGPaqkcqGGSaalaSqaaiabdwgaLjabg2da9iabigdaXaqaaiabdweafbqdcqGHpis1aaWcbaGaem4zaCMaeyypa0JaeGymaedabaGaem4raCeaniabg+GivdaaleaacqWGQbGAcqGH9aqpcqaIXaqmaeaacqWGkbGsa0Gaey4dIunaaaaaaa@DD76@

where **Ω**_**j **_= (ηjg02
 MathType@MTEF@5@5@+=feaafiart1ev1aaatCvAUfKttLearuWrP9MDH5MBPbIqV92AaeXatLxBI9gBaebbnrfifHhDYfgasaacH8akY=wiFfYdH8Gipec8Eeeu0xXdbba9frFj0=OqFfea0dXdd9vqai=hGuQ8kuc9pgc9s8qqaq=dirpe0xb9q8qiLsFr0=vr0=vr0dc8meaabaqaciaacaGaaeqabaqabeGadaaakeaaiiGacqWF3oaAdaqhaaWcbaGaemOAaOMaem4zaCMaeGimaadabaGaeGOmaidaaaaa@3320@, *c*_*j*_), *j *= study, *g *= gene, *e *= experiment, *s *= slide.

#### Prior distributions

We assigned as uninformative prior distributions as possible to the parameters of Models (1) and (2) that still resulted in convergence of the models. For the parameter *p*, we assigned a non-informative Uniform(0, l) distribution. The prior distributions of the slide effect and experiment effect variance parameters, φjg2
 MathType@MTEF@5@5@+=feaafiart1ev1aaatCvAUfKttLearuWrP9MDH5MBPbIqV92AaeXatLxBI9gBaebbnrfifHhDYfgasaacH8akY=wiFfYdH8Gipec8Eeeu0xXdbba9frFj0=OqFfea0dXdd9vqai=hGuQ8kuc9pgc9s8qqaq=dirpe0xb9q8qiLsFr0=vr0=vr0dc8meaabaqaciaacaGaaeqabaqabeGadaaakeaaiiGacqWFgpGzdaqhaaWcbaGaemOAaOMaem4zaCgabaGaeGOmaidaaaaa@323F@ and σjg2
 MathType@MTEF@5@5@+=feaafiart1ev1aaatCvAUfKttLearuWrP9MDH5MBPbIqV92AaeXatLxBI9gBaebbnrfifHhDYfgasaacH8akY=wiFfYdH8Gipec8Eeeu0xXdbba9frFj0=OqFfea0dXdd9vqai=hGuQ8kuc9pgc9s8qqaq=dirpe0xb9q8qiLsFr0=vr0=vr0dc8meaabaqaciaacaGaaeqabaqabeGadaaakeaaiiGacqWFdpWCdaqhaaWcbaGaemOAaOMaem4zaCgabaGaeGOmaidaaaaa@3249@, respectively, required some information from the data in order for the models to converge. We assigned the following prior distributions for these parameters (see also [17,23,24]):

φjg2~kφ˜j2/χk2σjg2~hσ˜j2/χh2
 MathType@MTEF@5@5@+=feaafiart1ev1aaatCvAUfKttLearuWrP9MDH5MBPbIqV92AaeXatLxBI9gBaebbnrfifHhDYfgasaacH8akY=wiFfYdH8Gipec8Eeeu0xXdbba9frFj0=OqFfea0dXdd9vqai=hGuQ8kuc9pgc9s8qqaq=dirpe0xb9q8qiLsFr0=vr0=vr0dc8meaabaqaciaacaGaaeqabaqabeGadaaakeaafaqadeGabaaabaacciGae8NXdy2aa0baaSqaaiabdQgaQjabdEgaNbqaaiabikdaYaaakiabc6ha+naalyaabaGaem4AaSMaf8NXdyMbaGaadaqhaaWcbaGaemOAaOgabaGaeGOmaidaaaGcbaGae83Xdm2aa0baaSqaaiabdUgaRbqaaiabikdaYaaaaaaakeaacqWFdpWCdaqhaaWcbaGaemOAaOMaem4zaCgabaGaeGOmaidaaOGaeiOFa43aaSGbaeaacqWGObaAcuWFdpWCgaacamaaDaaaleaacqWGQbGAaeaacqaIYaGmaaaakeaacqWFhpWydaqhaaWcbaGaemiAaGgabaGaeGOmaidaaaaaaaaaaa@4EDF@

Here, φ˜j2
 MathType@MTEF@5@5@+=feaafiart1ev1aaatCvAUfKttLearuWrP9MDH5MBPbIqV92AaeXatLxBI9gBaebbnrfifHhDYfgasaacH8akY=wiFfYdH8Gipec8Eeeu0xXdbba9frFj0=OqFfea0dXdd9vqai=hGuQ8kuc9pgc9s8qqaq=dirpe0xb9q8qiLsFr0=vr0=vr0dc8meaabaqaciaacaGaaeqabaqabeGadaaakeaaiiGacuWFgpGzgaacamaaDaaaleaacqWGQbGAaeaacqaIYaGmaaaaaa@30F7@ and σ˜j2
 MathType@MTEF@5@5@+=feaafiart1ev1aaatCvAUfKttLearuWrP9MDH5MBPbIqV92AaeXatLxBI9gBaebbnrfifHhDYfgasaacH8akY=wiFfYdH8Gipec8Eeeu0xXdbba9frFj0=OqFfea0dXdd9vqai=hGuQ8kuc9pgc9s8qqaq=dirpe0xb9q8qiLsFr0=vr0=vr0dc8meaabaqaciaacaGaaeqabaqabeGadaaakeaaiiGacuWFdpWCgaacamaaDaaaleaacqWGQbGAaeaacqaIYaGmaaaaaa@3101@ are scale parameters of the inverse chi-squared distribution obtained from the data. φ˜j2
 MathType@MTEF@5@5@+=feaafiart1ev1aaatCvAUfKttLearuWrP9MDH5MBPbIqV92AaeXatLxBI9gBaebbnrfifHhDYfgasaacH8akY=wiFfYdH8Gipec8Eeeu0xXdbba9frFj0=OqFfea0dXdd9vqai=hGuQ8kuc9pgc9s8qqaq=dirpe0xb9q8qiLsFr0=vr0=vr0dc8meaabaqaciaacaGaaeqabaqabeGadaaakeaaiiGacuWFgpGzgaacamaaDaaaleaacqWGQbGAaeaacqaIYaGmaaaaaa@30F7@ is computed as follows:

φ˜j2=1G(∑Se−1)∑g=1G∑e=1E∑s=1Se(yjgse−yjg.e)2,
 MathType@MTEF@5@5@+=feaafiart1ev1aaatCvAUfKttLearuWrP9MDH5MBPbIqV92AaeXatLxBI9gBaebbnrfifHhDYfgasaacH8akY=wiFfYdH8Gipec8Eeeu0xXdbba9frFj0=OqFfea0dXdd9vqai=hGuQ8kuc9pgc9s8qqaq=dirpe0xb9q8qiLsFr0=vr0=vr0dc8meaabaqaciaacaGaaeqabaqabeGadaaakeaaiiGacuWFgpGzgaacamaaDaaaleaacqWGQbGAaeaacqaIYaGmaaGccqGH9aqpdaWcaaqaaiabigdaXaqaaiabdEeahnaabmaabaWaaabqaeaacqWGtbWudaWgaaWcbaGaemyzaugabeaakiabgkHiTiabigdaXaWcbeqab0GaeyyeIuoaaOGaayjkaiaawMcaaaaadaaeWbqaamaaqahabaWaaabCaeaadaqadaqaaiabdMha5naaBaaaleaacqWGQbGAcqWGNbWzcqWGZbWCcqWGLbqzaeqaaOGaeyOeI0IaemyEaK3aaSbaaSqaaiabdQgaQjabdEgaNjabc6caUiabdwgaLbqabaaakiaawIcacaGLPaaadaahaaWcbeqaaiabikdaYaaaaeaacqWGZbWCcqGH9aqpcqaIXaqmaeaacqWGtbWudaWgaaadbaGaemyzaugabeaaa0GaeyyeIuoaaSqaaiabdwgaLjabg2da9iabigdaXaqaaiabdweafbqdcqGHris5aaWcbaGaem4zaCMaeyypa0JaeGymaedabaGaem4raCeaniabggHiLdGccqGGSaalaaa@642B@

where *y*_*jg*.*e *_is the log-expression ratio averaged over the slides within each experiment:

yjg.e=1Se∑s=1Seyjgse.
 MathType@MTEF@5@5@+=feaafiart1ev1aaatCvAUfKttLearuWrP9MDH5MBPbIqV92AaeXatLxBI9gBaebbnrfifHhDYfgasaacH8akY=wiFfYdH8Gipec8Eeeu0xXdbba9frFj0=OqFfea0dXdd9vqai=hGuQ8kuc9pgc9s8qqaq=dirpe0xb9q8qiLsFr0=vr0=vr0dc8meaabaqaciaacaGaaeqabaqabeGadaaakeaacqWG5bqEdaWgaaWcbaGaemOAaOMaem4zaCMaeiOla4Iaemyzaugabeaakiabg2da9maalaaabaGaeGymaedabaGaem4uam1aaSbaaSqaaiabdwgaLbqabaaaaOWaaabCaeaacqWG5bqEdaWgaaWcbaGaemOAaOMaem4zaCMaem4CamNaemyzaugabeaaaeaacqWGZbWCcqGH9aqpcqaIXaqmaeaacqWGtbWudaWgaaadbaGaemyzaugabeaaa0GaeyyeIuoakiabc6caUaaa@485C@

The scale parameter for σjg2
 MathType@MTEF@5@5@+=feaafiart1ev1aaatCvAUfKttLearuWrP9MDH5MBPbIqV92AaeXatLxBI9gBaebbnrfifHhDYfgasaacH8akY=wiFfYdH8Gipec8Eeeu0xXdbba9frFj0=OqFfea0dXdd9vqai=hGuQ8kuc9pgc9s8qqaq=dirpe0xb9q8qiLsFr0=vr0=vr0dc8meaabaqaciaacaGaaeqabaqabeGadaaakeaaiiGacqWFdpWCdaqhaaWcbaGaemOAaOMaem4zaCgabaGaeGOmaidaaaaa@3249@ is similarly produced as follows:

σ˜j2=1G(E−1)∑g=1G∑e=1E(yjg.e−yjg..)2,
 MathType@MTEF@5@5@+=feaafiart1ev1aaatCvAUfKttLearuWrP9MDH5MBPbIqV92AaeXatLxBI9gBaebbnrfifHhDYfgasaacH8akY=wiFfYdH8Gipec8Eeeu0xXdbba9frFj0=OqFfea0dXdd9vqai=hGuQ8kuc9pgc9s8qqaq=dirpe0xb9q8qiLsFr0=vr0=vr0dc8meaabaqaciaacaGaaeqabaqabeGadaaakeaaiiGacuWFdpWCgaacamaaDaaaleaacqWGQbGAaeaacqaIYaGmaaGccqGH9aqpdaWcaaqaaiabigdaXaqaaiabdEeahjabcIcaOiabdweafjabgkHiTiabigdaXiabcMcaPaaadaaeWbqaamaaqahabaWaaeWaaeaacqWG5bqEdaWgaaWcbaGaemOAaOMaem4zaCMaeiOla4IaemyzaugabeaakiabgkHiTiabdMha5naaBaaaleaacqWGQbGAcqWGNbWzcqGGUaGlcqGGUaGlaeqaaaGccaGLOaGaayzkaaWaaWbaaSqabeaacqaIYaGmaaaabaGaemyzauMaeyypa0JaeGymaedabaGaemyraueaniabggHiLdaaleaacqWGNbWzcqGH9aqpcqaIXaqmaeaacqWGhbWra0GaeyyeIuoakiabcYcaSaaa@574B@

where *y*_*jg.. *_is the log-expression ratio averaged over both slides and experiments. We assigned 3 degrees of freedom in each study for φjg2
 MathType@MTEF@5@5@+=feaafiart1ev1aaatCvAUfKttLearuWrP9MDH5MBPbIqV92AaeXatLxBI9gBaebbnrfifHhDYfgasaacH8akY=wiFfYdH8Gipec8Eeeu0xXdbba9frFj0=OqFfea0dXdd9vqai=hGuQ8kuc9pgc9s8qqaq=dirpe0xb9q8qiLsFr0=vr0=vr0dc8meaabaqaciaacaGaaeqabaqabeGadaaakeaaiiGacqWFgpGzdaqhaaWcbaGaemOAaOMaem4zaCgabaGaeGOmaidaaaaa@323F@ and σjg2
 MathType@MTEF@5@5@+=feaafiart1ev1aaatCvAUfKttLearuWrP9MDH5MBPbIqV92AaeXatLxBI9gBaebbnrfifHhDYfgasaacH8akY=wiFfYdH8Gipec8Eeeu0xXdbba9frFj0=OqFfea0dXdd9vqai=hGuQ8kuc9pgc9s8qqaq=dirpe0xb9q8qiLsFr0=vr0=vr0dc8meaabaqaciaacaGaaeqabaqabeGadaaakeaaiiGacqWFdpWCdaqhaaWcbaGaemOAaOMaem4zaCgabaGaeGOmaidaaaaa@3249@, i.e. *h *= *k *= 3, so that the distribution is as uninformative as possible (see also Results and Discussion: Sensitivity analysis). The remaining parameters were assigned the following prior distributions, which were as uninformative as possible while still allowing the models to converge.

ηg02~as12/χa2c~bs22/χb2
 MathType@MTEF@5@5@+=feaafiart1ev1aaatCvAUfKttLearuWrP9MDH5MBPbIqV92AaeXatLxBI9gBaebbnrfifHhDYfgasaacH8akY=wiFfYdH8Gipec8Eeeu0xXdbba9frFj0=OqFfea0dXdd9vqai=hGuQ8kuc9pgc9s8qqaq=dirpe0xb9q8qiLsFr0=vr0=vr0dc8meaabaqaciaacaGaaeqabaqabeGadaaakeaafaqadeGabaaabaacciGae83TdG2aa0baaSqaaiabdEgaNjabicdaWaqaaiabikdaYaaakiabc6ha+naalyaabaGaemyyaeMaem4Cam3aa0baaSqaaiabigdaXaqaaiabikdaYaaaaOqaaiab=D8aJnaaDaaaleaacqWGHbqyaeaacqaIYaGmaaaaaaGcbaGaem4yamMaeiOFa43aaSGbaeaacqWGIbGycqWGZbWCdaqhaaWcbaGaeGOmaidabaGaeGOmaidaaaGcbaGae83Xdm2aa0baaSqaaiabdkgaIbqaaiabikdaYaaaaaaaaaaa@484D@

The degrees of freedom and scale parameters *a*, s12
 MathType@MTEF@5@5@+=feaafiart1ev1aaatCvAUfKttLearuWrP9MDH5MBPbIqV92AaeXatLxBI9gBaebbnrfifHhDYfgasaacH8akY=wiFfYdH8Gipec8Eeeu0xXdbba9frFj0=OqFfea0dXdd9vqai=hGuQ8kuc9pgc9s8qqaq=dirpe0xb9q8qiLsFr0=vr0=vr0dc8meaabaqaciaacaGaaeqabaqabeGadaaakeaacqWGZbWCdaqhaaWcbaGaeGymaedabaGaeGOmaidaaaaa@302A@, respectively, were assigned so that the prior mean of ηg02
 MathType@MTEF@5@5@+=feaafiart1ev1aaatCvAUfKttLearuWrP9MDH5MBPbIqV92AaeXatLxBI9gBaebbnrfifHhDYfgasaacH8akY=wiFfYdH8Gipec8Eeeu0xXdbba9frFj0=OqFfea0dXdd9vqai=hGuQ8kuc9pgc9s8qqaq=dirpe0xb9q8qiLsFr0=vr0=vr0dc8meaabaqaciaacaGaaeqabaqabeGadaaakeaaiiGacqWF3oaAdaqhaaWcbaGaem4zaCMaeGimaadabaGaeGOmaidaaaaa@31C3@ was 1 with variance 0.1, and *b*, s22
 MathType@MTEF@5@5@+=feaafiart1ev1aaatCvAUfKttLearuWrP9MDH5MBPbIqV92AaeXatLxBI9gBaebbnrfifHhDYfgasaacH8akY=wiFfYdH8Gipec8Eeeu0xXdbba9frFj0=OqFfea0dXdd9vqai=hGuQ8kuc9pgc9s8qqaq=dirpe0xb9q8qiLsFr0=vr0=vr0dc8meaabaqaciaacaGaaeqabaqabeGadaaakeaacqWGZbWCdaqhaaWcbaGaeGOmaidabaGaeGOmaidaaaaa@302C@ were assigned so that the prior mean of *c *was 100 with variance 10,000. For a description of the prior distribution for τg2
 MathType@MTEF@5@5@+=feaafiart1ev1aaatCvAUfKttLearuWrP9MDH5MBPbIqV92AaeXatLxBI9gBaebbnrfifHhDYfgasaacH8akY=wiFfYdH8Gipec8Eeeu0xXdbba9frFj0=OqFfea0dXdd9vqai=hGuQ8kuc9pgc9s8qqaq=dirpe0xb9q8qiLsFr0=vr0=vr0dc8meaabaqaciaacaGaaeqabaqabeGadaaakeaaiiGacqWFepaDdaqhaaWcbaGaem4zaCgabaGaeGOmaidaaaaa@30EE@, see the Methods section: Standardized expression integration model: prior distributions for inter-study variation.

#### Full conditional posterior distributions

Each parameter was sampled from the full conditional posterior distributions by an MCMC algorithm using the WinBUGS software [61]. We used 5,000 iterations for all analyses, which was more than sufficient. Further MCMC implementation details can be found in Conlon *et al*. [15].

#### WinBUGS running time

The WinBUGS running time ranged from approximately 7 minutes for 3,000 genes and two studies for the PI model to 2.41 hours for 20,000 genes and five studies for the SEI model, for 5,000 iterations, using a personal computer with an Intel Core Duo T2500 2.0 GHz Processor. Table 6 details running times in seconds for the number of genes: 3,000, 10,000, 20,000, for two and five studies and 5,000 iterations for both the PI and SEI models.

**Table 6:** Runtime values for the WinBUGS MCMC implementation. Runtime values in seconds for 5,000 iterations of the WinBUGS MCMC implementation of SEI and PI models for various numbers of genes and studies, using a personal computer with an Intel Core Duo T2500 2.0 GHz Processor.

**Runtime (Seconds)**			
	**Number of Genes**		
	
**Two Studies**	**3,000**	**10,000**	**20,000**

**SEI model**	561	2,652	4,621
**PI model**	415	1,947	3,375

**Five Studies**			

**SEI model**	1,511	4,981	8,679
**PI model**	1,204	4,287	8,008

## Availability and requirements

The WinBUGS code for implementing the models is included in the Supplemental Files (see Additional files 2 and 3).

Operating system: Windows 98 or later.

Other requirements: WinBUGS software version 1.4 or later [61].

License: free.

## Authors' contributions

All authors contributed to development of the methodology. EMC and JJS contributed to writing the computer code and writing the manuscript.

## Supplementary Material

Additional File 1 **Supplemental Figures**. containing Figures for the Sensitivity Analysis Results. Click here for file

Additional File 2 **WinBUGS code for the SEI model**. containing the WinBUGS code for the SEI model. Click here for file

Additional File 3 **WinBUGS code for the PI model**. containing the WinBUGS code for the PI model. Click here for file
