# Supplementary material for: Bayesian meta-analysis models for microarray data: a comparative study
Source: BMC Bioinformatics. 2007 Mar 7;8:80. doi: 10.1186/1471-2105-8-80 (PMC1851021; doi:10.1186/1471-2105-8-80)

Supplemental Figures

Bayesian meta-analysis models for microarray data: a comparative study

Erin M Conlon[§], Joon J Song, Anna Liu
[§]Corresponding author: econlon@mathstat.umass.edu

Figure S1 - Sensitivity analysis results for the two-study simulation data

Sensitivity analysis results for 3 and 40 degrees of freedom for the prior distributions of the slide and experiment variation parameters, ϕ^2_{jg} and σ^2_{jg}, respectively, for the two-study simulation data set with medium mean τ^2_g = 0.3 (differentially expressed); 0.03 (non-differentially expressed) and simulated percent differentially expressed genes p_s = 10%. a) True integration-driven discovery rate (*t*IDR) versus threshold values of posterior probability of differential expression $\gamma \geq 0.50$, for the standardized expression integration model (blue circles) and probability integration model (black diamonds); b) True integration-driven revision rate (*t*IRR) versus threshold values of posterior probability of differential expression $\gamma \geq 0.50$, for the standardized expression integration model (blue circles) and probability integration model (black diamonds); c) The maximum number of true discovered genes versus posterior expected false discovery rate (*pe*FDR) for the standardized expression integration model (blue circles), probability integration model (black diamonds), individual analyses of Study 1 (red checks) and Study 2 (green triangles).

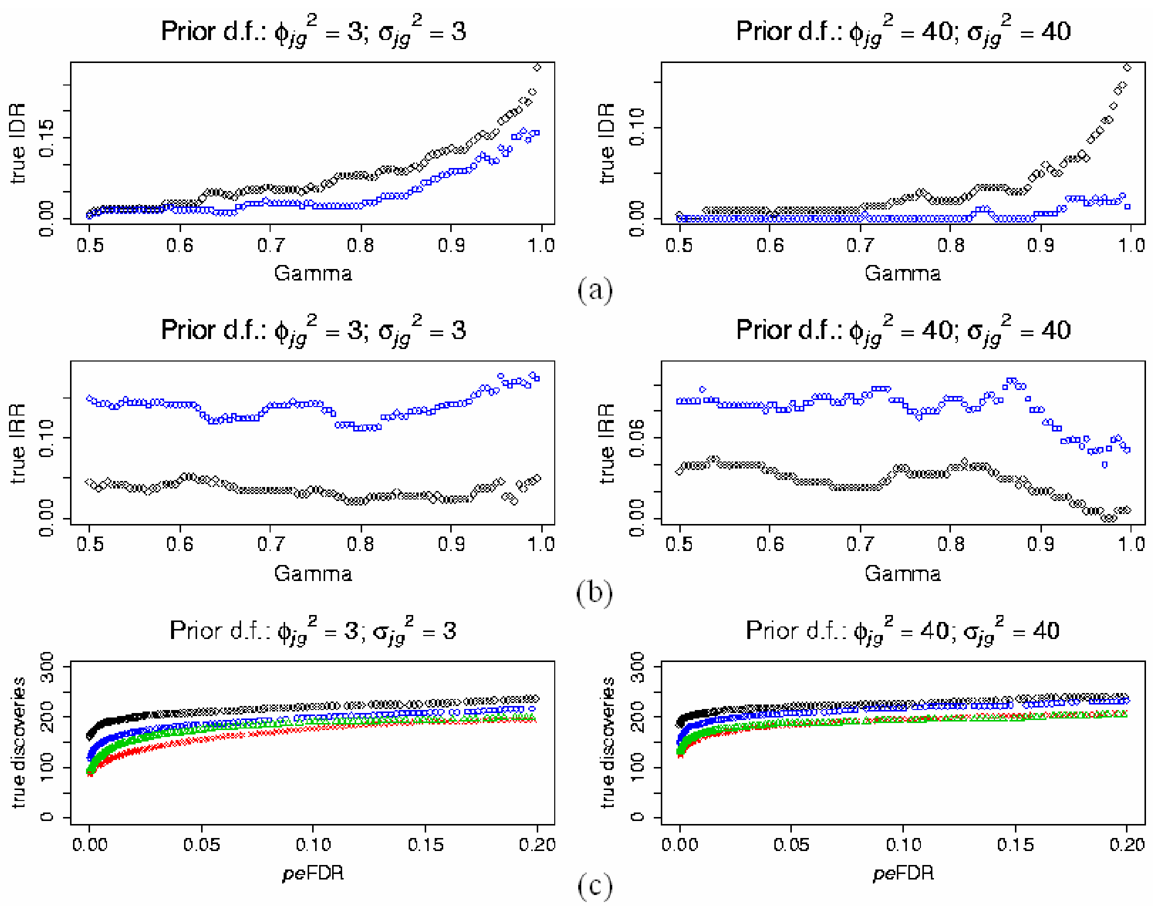

Figure S2 - Sensitivity analysis results for the five-study simulation data

Sensitivity analysis results for 3 and 40 degrees of freedom for the prior distributions of the slide and experiment variation parameters, ϕ_{jg}^2 and σ_{jg}^2, respectively, for the five-study simulation data set with medium mean $\tau_g^2 = 0.3$ (differentially expressed); 0.03 (non-differentially expressed) and simulated percent differentially expressed genes p_s = 10%. a) True integration-driven discovery rate (*t*IDR) versus threshold values of posterior probability of differential expression $\gamma \geq 0.50$, for the standardized expression integration model (blue circles) and probability integration model (black diamonds); b) True integration-driven revision rate (*t*IRR) versus threshold values of posterior probability of differential expression $\gamma \geq 0.50$, for the standardized expression integration model (blue circles) and probability integration model (black diamonds); c) The maximum number of true discovered genes versus posterior expected false discovery rate (*pe*FDR) for the standardized expression integration model (blue circles), probability integration model (black diamonds), individual analyses of Study 1 (red checks), Study 2 (green triangles), Study 3 (turquoise pluses), Study 4 (pink inverted triangles) and Study 5 (gold stars).

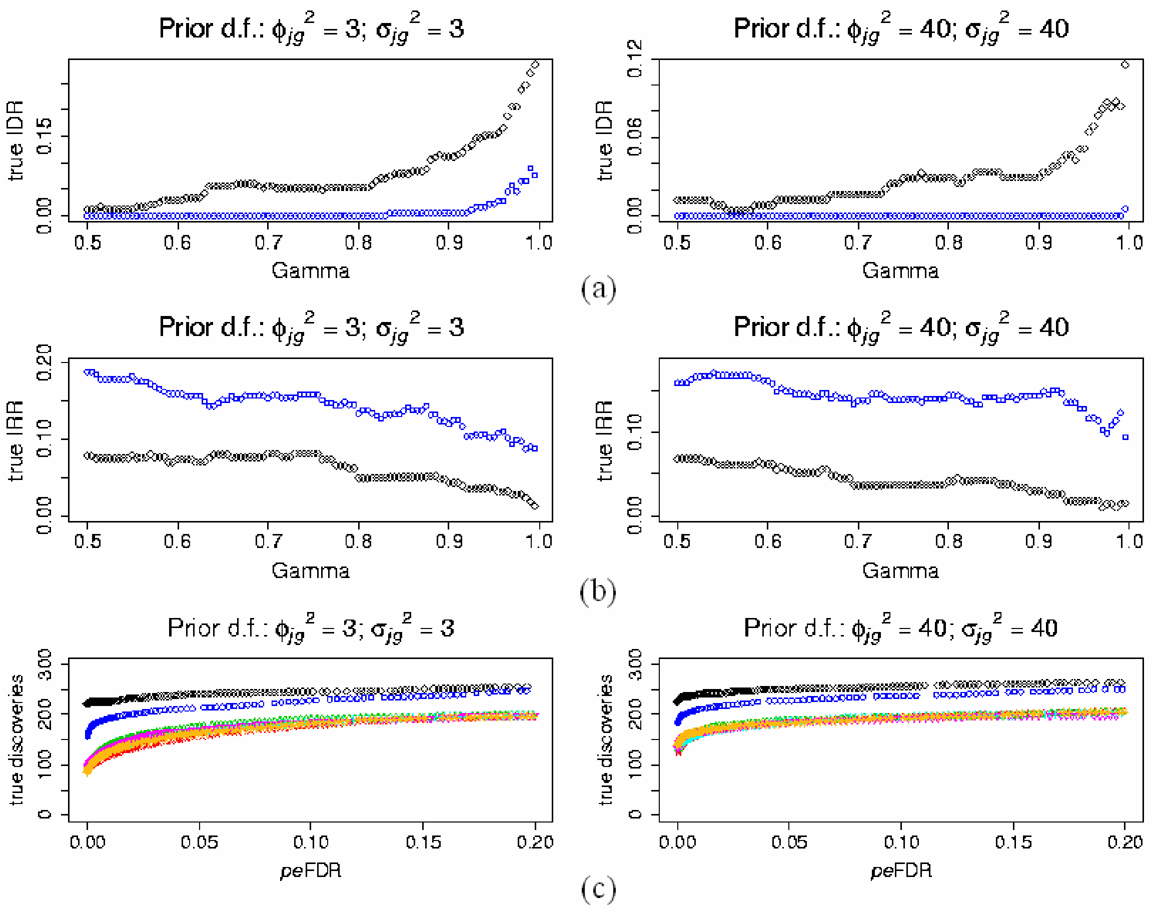

Figure S3 - Sensitivity analysis results for the biological data

Sensitivity analysis results for 3 and 40 degrees of freedom for the prior distributions of the slide and experiment variation parameters, ϕ_{jg}^2 and σ_{jg}^2, respectively, for the *B. subtilis* mutant and induction biological study data. a) Integration-driven discovery rate (IDR) versus threshold values of posterior probability of differential expression $\gamma \geq 0.50$, for the standardized expression integration model (blue circles) and probability integration model (black diamonds); b) Integration-driven revision rate (IRR) versus threshold values of posterior probability of differential expression $\gamma \geq 0.50$, for the standardized expression integration model (blue circles) and probability integration model (black diamonds); c) The maximum number of differentially expressed genes versus posterior expected false discovery rate (*pe*FDR) for the standardized expression integration model (blue circles), probability integration model (black diamonds), individual analyses of *B. subtilis* mutant study (red checks) and induction study (green triangles).

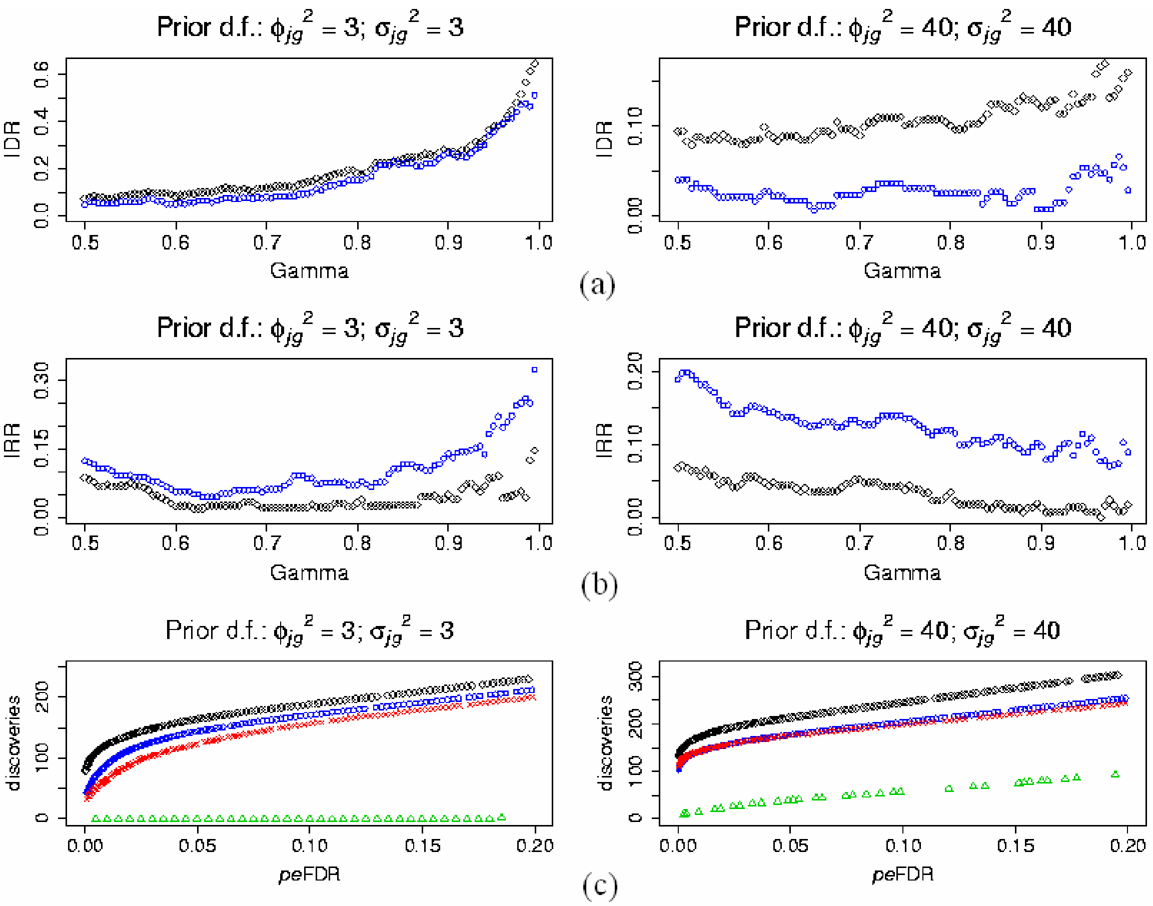

Supplement: Additional File 1 — Supplemental Figures. containing Figures for the Sensitivity Analysis Results. [file 1471-2105-8-80-S1.pdf]
